# Legume-specific recruitment of rhizobia by hyphae of arbuscular mycorrhizal fungi

**DOI:** 10.1093/ismejo/wraf100

**Published:** 2025-05-21

**Authors:** Jiadong He, Judith Van Dingenen, Sofie Goormachtig, Maryline Calonne-Salmon, Stéphane Declerck

**Affiliations:** Laboratory of Mycology, Earth and Life Institute, Université Catholique de Louvain-UCLouvain, Croix du Sud 2, L7.05.06, 1348 Louvain-la-Neuve, Belgium; Department of Plant Biotechnology and Bioinformatics, Ghent University, Technologiepark 71, 9052 Ghent, Belgium; Center for Plant Systems Biology, VIB, Technologiepark 71, 9052 Ghent, Belgium; Department of Plant Biotechnology and Bioinformatics, Ghent University, Technologiepark 71, 9052 Ghent, Belgium; Center for Plant Systems Biology, VIB, Technologiepark 71, 9052 Ghent, Belgium; Laboratory of Mycology, Earth and Life Institute, Université Catholique de Louvain-UCLouvain, Croix du Sud 2, L7.05.06, 1348 Louvain-la-Neuve, Belgium; Laboratory of Mycology, Earth and Life Institute, Université Catholique de Louvain-UCLouvain, Croix du Sud 2, L7.05.06, 1348 Louvain-la-Neuve, Belgium

**Keywords:** arbuscular *mycorrhizal* (AM) fungi, common mycorrhizal network (CMN), flavonoids, nitrogen fixation, nodulation, rhizobia

## Abstract

The legume-rhizobia symbiosis possesses great potential for sustainable agriculture because of its ability to fix atmospheric nitrogen, reducing crop dependence on nitrogen fertilizers. Rhizobia recognize the host legume through flavonoids released by the roots. These signals are detected by bacteria typically over a few millimeters. Recent research has shown that arbuscular mycorrhizal fungi extend this recognition beyond 15 cm by transporting flavonoids along their hyphae. In soil, common mycorrhizal networks (CMNs) linking plants are formed by arbuscular mycorrhizal fungi. We hypothesized that such networks linking different legumes can transmit host-specific signals, guiding rhizobia to their appropriate hosts. Using *in vitro* and greenhouse microcosms, we linked *Medicago truncatula* and *Glycine max* via a CMN of *Rhizophagus irregularis* and inoculated GFP-labeled *Sinorhizobium meliloti* and mCherry-labeled *Bradyrhizobium diazoefficiens* on the hyphae. *S. meliloti* preferentially migrated towards *M. truncatula*, whereas *B. diazoefficiens* preferentially migrated towards *G. max* (155 ± 8 and 13 ± 3 nodules, respectively). This was confirmed in the greenhouse with a higher concentration of *S. meliloti* (2.1–2.5 × 10^5^ CFU·g^−1^) near *M. truncatula* and a higher concentration of *B. diazoefficiens* (1.5–1.6 × 10^5^ CFU·g^−1^) near *G. max* (71–82 and 15–18 nodules, respectively). Metabolomics revealed host-specific flavonoids in hyphal exudates: *M. truncatula*-connected hyphae released DL-liquiritigenin, naringenin, sakuranetin, and 3,7-dimethylquercetin, whereas *G. max*-connected hyphae released daidzin, 6"-O-malonyldaidzin, irilone, and erylatissin A. These findings establish that common mycorrhizal networks constitute a “navigation system”, using chemical signals to orient rhizobia towards their specific hosts, thereby improving nodulation with potential applications in agriculture.

## Introduction

Nitrogen-fixing bacteria (collectively known as rhizobia) can establish beneficial interactions with legumes and fix atmospheric dinitrogen (N_2_) by forming a specialized organ, the nodule [1]. However, they can only fix N_2_ when they form nodules with roots of compatible leguminous plants [2, 3]. For example, *Medicago truncatula* forms indeterminate nodules with *Sinorhizobium meliloti*, whereas *Glycine max* forms determinate nodules with *Bradyrhizobium diazoefficiens* [4, 5].

Nodulating rhizobia exhibit limited mobility in soil and typically originate from the soil environment rather than from seeds [6]. They are attracted by specific compounds (flavonoids) released over a short distance (a few mm) by root epidermal cells and root hairs [7]. As a result, rhizobia separated from their hosts by a long distance are unable to detect the host. In addition, the presence of air-filled gaps between soil aggregates prevents rhizobia from moving toward the roots using flagella without the aid of flowing water or other vectors [8, 9].

Previous research demonstrated that *S. meliloti* can migrate to the roots of legumes (i.e. *M. truncatula*) from long distances (several cm) via the surface of the hyphae of root-associated arbuscular mycorrhizal (AM) fungi, leading to nodulation [10]. Because the vast majority of legumes are hosts to AM fungi (their hyphae provide a hospitable environment for rhizobia and the mycelium of these obligate root symbionts is extensive and abundant [11, 12], spreads widely in the soil – from 82 to 111 m·cm^−3^ in grassland and from 52 to 81 m·cm^−3^ in ungrazed pasture) [13], these belowground fungi represent an important indirect route for legumes to recruit root-nodulating bacteria.

In soils, individual plants can be interconnected by common mycorrhizal networks (CMNs) formed by AM fungal hyphae [14–16]. These networks have been explored for their potential roles in mediating interactions between connected plants, including the transfer of carbon and nutrients [17], and the transmission of signals involved in processes such as defense [18, 19]. However, the extent, net directionality, and ecological significance of these functions, are subjects of ongoing research and critical debate. Recent analyses have urged caution against overinterpretation and highlighted the influence of methodological choices and potential citation biases in some areas of CMN research [20, 21]. Nonetheless, experimental studies have provided evidence that certain signaling molecules can be transported between plants via CMNs under specific conditions, for instance, in the context of induced plant defense against herbivores [22–24]. These experimental observations of the movement of signaling molecules between plants via mycelial connections suggest that CMNs could also facilitate the transport of host-specific signals, such as flavonoids, involved in the recruitment of rhizobia.

It has been demonstrated that *S. meliloti,* plated on AM fungal hyphae linking a legume (*M. truncatula*) to a non-legume (*Solanum tuberosum*) by a CMN, migrated preferentially towards the legume suggesting the chemoattraction by specific signals transported and released by the AM fungus connected to the legume [10]. This migration pattern was confirmed by a metabolomics study revealing the presence of eight specific flavonoids released by AM fungal hyphae linked to *M. truncatula* [10]*.* However, it is not known whether signals transmitted by two different legumes connected by a CMN can enable rhizobia to specifically recognize their compatible host and migrate towards it selectively.

In the present study we conducted a series of *in vitro* and greenhouse experiments with two stable fluorescent rhizobia (*S. meliloti* pHC60-GFP and *B. diazoefficiens* mChe-1) specific to *M. truncatula* and *G. max*, respectively, connected by a CMN of *Rhizophagus irregularis* MUCL 41833, for studying preferential migration. We first confirmed *in vitro* that *S. meliloti* form nodules with *M. truncatula* and not *G. max* and conversely that *B. diazoefficiens* from nodules with *G. max* and not with *M. truncatula*. Next, we conducted a comparative metabolomics study on the growth media containing AM fungal hyphae connected to *M. truncatula* or *G. max* in a compartmented system to reveal the presence and potential differences in flavonoids content. Finally, we carried out an *in vitro* and a pot microcosm experiment with the two leguminous plants connected by a CMN of the AM fungus to explore whether *S. meliloti* pHC60-GFP and *B. diazoefficiens* mChe-1 show selective tropism towards their respective plant hosts.

We show that CMN connecting two different legumes species allow rhizobia to recognize and preferentially migrate towards their specific host and form nodules. This preferential directional migration is probably guided by host-specific flavonoid signals, as shown by comparative metabolomics analysis, which reveals differences between hyphal exudates connected to the two legumes species.

## Materials and methods

### Biological material

Experiments were conducted using *Rhizophagus irregularis* MUCL 41833 (GINCO, Belgium), maintained *in vitro* on Ri T-DNA transformed carrot (*Daucus carota* L.) roots as described previously [25]. *Sinorhizobium meliloti* 2011 pHC60-GFP and *Bradyrhizobium diazoefficiens* mChe-1, tagged with GFP [26] and mCherry [27] respectively, were provided by VIB-UGent Center for Plant Systems Biology (Belgium) and cultured in yeast extract broth (YEB) medium with appropriate antibiotics [10]. Seeds of *Medicago truncatula* L. cv. Jemalong A17, *Glycine max* (L.) Merr., and *Plantago lanceolata* L. were sourced from SARDI (Australia), VIB-UGent (Belgium), and ECOSEM (Belgium), respectively, and germinated on sterilized Modified Strullu-Romand medium (MSR) medium after surface disinfection [28]. See Supplementary Materials for detailed culturing and preparation methods.

### Growth and nodulation of *S. meliloti* and *B. diazoefficiens* on *M. truncatula* and *G. max*

**Figure 1:**
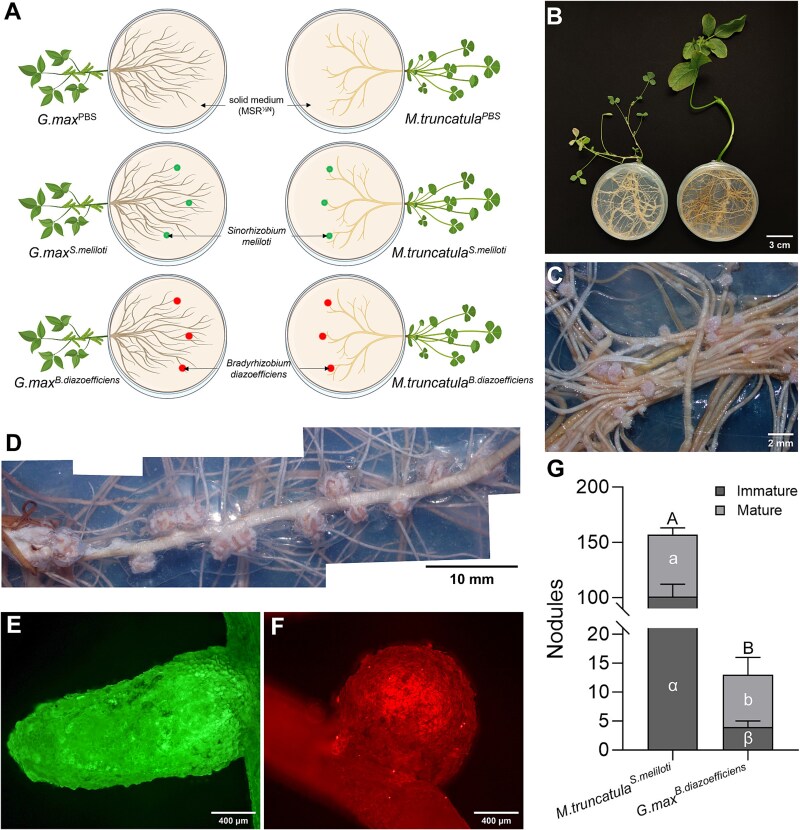
Experimental setup and evaluation of nodulation of *M. truncatula* and *G. max in vitro*. (A) Schematic representation of the mono-compartmented Petri plate system, with distinct representations for *S. meliloti* and *B. diazoefficiens* inoculum. (B) Photograph of the mono-compartmented Petri plate system with *M. truncatula* on the left and *G. max* on the right. (C) Nodules formed on *M. truncatula* roots inoculated with *S. meliloti*. (D) Nodules formed on *G. max* roots inoculated with *B. diazoefficiens*. (E) Epifluorescence microscopy images of an indeterminate immature nodule on a root of *M. truncatula*. (F) Epifluorescence microscopy images of a determinate mature nodule formed on a root of *G. max*. (G) Number of immature and mature nodules formed on roots of *M. truncatula* inoculated with *S. meliloti* (*M.truncatula^S.meliloti^* treatment) and on roots of *G. max* inoculated with *B. diazoefficiens* (*G.max^B. diazoefficiens^* treatment), respectively, six weeks post-inoculation (*n* = 6 biological replicates). Different Greek letters (α, β), lowercase letters (a, b), and uppercase letters (A, B), respectively, indicate significant differences of immature, mature, and total nodule numbers between treatments (Student’s *t*-test, *P* ≤ 0.05). No nodules were observed in the *M. truncatula^B.diazoefficiens^* and *G.max^S. meliloti^* treatments (data not shown).

Seven-days-old *G. max* and *M. truncatula* plants were grown in Petri plates (90 mm diameter) on MSR^min½N^ medium (MSR medium lacking sucrose and vitamins (MSR^min^)), containing half the normal N concentration (1.99 mM)) [10]. Six treatments were established: *G. max* or *M. truncatula* inoculated with *S. meliloti*, *B. diazoefficiens* (150 μl, 9 × 10^5^ CFU·ml^−1^) or PBS (the controls), with six replicates per treatment (Fig. 1A, B). After 6 weeks, nodules were counted and classified by color (mature: pink/red; immature: white) and shape (determinate: spherical; indeterminate: elongated) [29, 30]. See Supplementary Materials for detailed inoculation and assessment methods.

### *In vitro* experimental design for analyzing flavonoids released by the ERM of *R. irregularis* connected to *G. max* or *M. truncatula*

**Figure 2:**
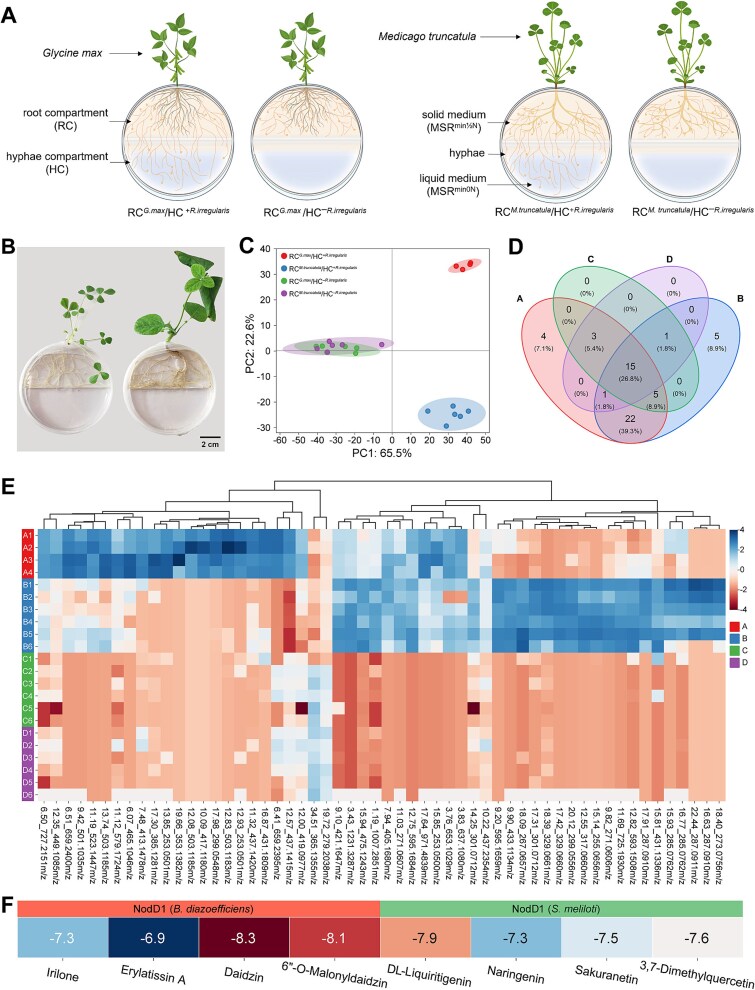
Experimental setup and analysis of extraradical mycelium (ERM) exudates in the *in vitro* bi-compartmented Petri plate system. (A) Schematic representation of the bi-compartmented Petri plate system used to analyze the transport and composition of ERM exudates. (B) Photograph of the bi-compartmented Petri plate system with *M. truncatula* on the left and *G. max* on the right. (C) Principal component analysis (PCA) plot based on compounds detected in the MSR^min0N^ medium collected from the hyphal compartment (HC), showing significant differences (ANOVA, *P* ≤ 0.01) between treatments. Ellipses represent the 95% confidence interval for each treatment. RC*^G.max^*/HC^+*R.irregularis*^ (*n* = 4 biological replicates); RC*^G.max^*/HC^–*R.irregularis*^ (*n* = 6 biological replicates); RC*^M.truncatula^*/HC^+*R.irregularis*^ (*n* = 6 biological replicates); RC*^M.truncatula^*/HC^–*R.irregularis*^ (*n* = 6 biological replicates). (D) Venn diagram summarizing the overlapping flavonoids between the four treatments. Counts of unique flavonoids are shown, with percentages relative to the total unique count in parentheses. A: RC*^G.max^*/HC^+*R.irregularis*^ (*n* = 4 biological replicates); B: RC*^M.truncatula^*/HC^+*R.irregularis*^ (*n* = 6 biological replicates); C: RC*^G.max^*/HC^–*R.irregularis*^ (*n* = 6 biological replicates); D: RC*^M.truncatula^*/HC^–*R.irregularis*^ (*n* = 6 biological replicates). (E) Heatmap displaying relative abundance profiles of flavonoids (columns) across different samples (rows, A1-D6). Different flavonoids were clustered using hierarchical clustering (complete linkage, Euclidean distance) to group similar profiles. A: RC*^G.max^*/HC^+*R.irregularis*^ (*n* = 4 biological replicates); B: RC*^M.truncatula^*/HC^+*R.irregularis*^ (*n* = 6 biological replicates); C: RC*^G.max^*/HC^–*R.irregularis*^ (*n* = 6 biological replicates); D: RC*^M.truncatula^*/HC^–*R.irregularis*^ (*n* = 6 biological replicates). (F) *In silico* binding studies between NodD1 protein of *B. diazoefficiens* (left four panels) and *S. meliloti* (right four panels) with flavonoids detected exclusively in RC*^G.max^*/HC^+*R.irregularis*^ and RC*^M.truncatula^*/HC^+*R.irregularis*^ treatments, respectively. Numbers in the panels represent binding affinities (kcal·Mol^−1^), where more negative values indicate stronger interactions.

Following a previous study [10], bi-compartmented Petri plates (90 mm diameter) were used with *G. max* or *M. truncatula* associated with *R. irregularis* in the root compartment (RC) containing 25 ml MSR^min½N^ medium, and with the AMF fungus extending alone in the hyphal compartment (HC) containing 10 ml liquid MSR^min0N^ medium (Fig. 2A, B). Four treatments were established: RC*^G.max^*/HC^+*R.irregularis*^, RC*^G.max^*/HC^–*R.irregularis*^, RC*^M.truncatula^*/HC^+*R.irregularis*^, and RC*^M.truncatula^*/HC^–*R.irregularis*^. At week 14, the liquid medium in the HC was collected for flavonoid analysis via UPLC-HRMS at the VIB Metabolomics Core Ghent, processed as previously described [10]. See Supplementary Materials for setup and analytical details.

### *In vitro* and greenhouse experimental designs with legumes linked by a CMN for mycelia-based migration assay of *S. meliloti* and *B. diazoefficiens*

The experimental design was adapted from [10], with modifications to accommodate the two plant species and two bacterial strains. See Supplementary Materials for detailed protocols.

**Figure 3:**
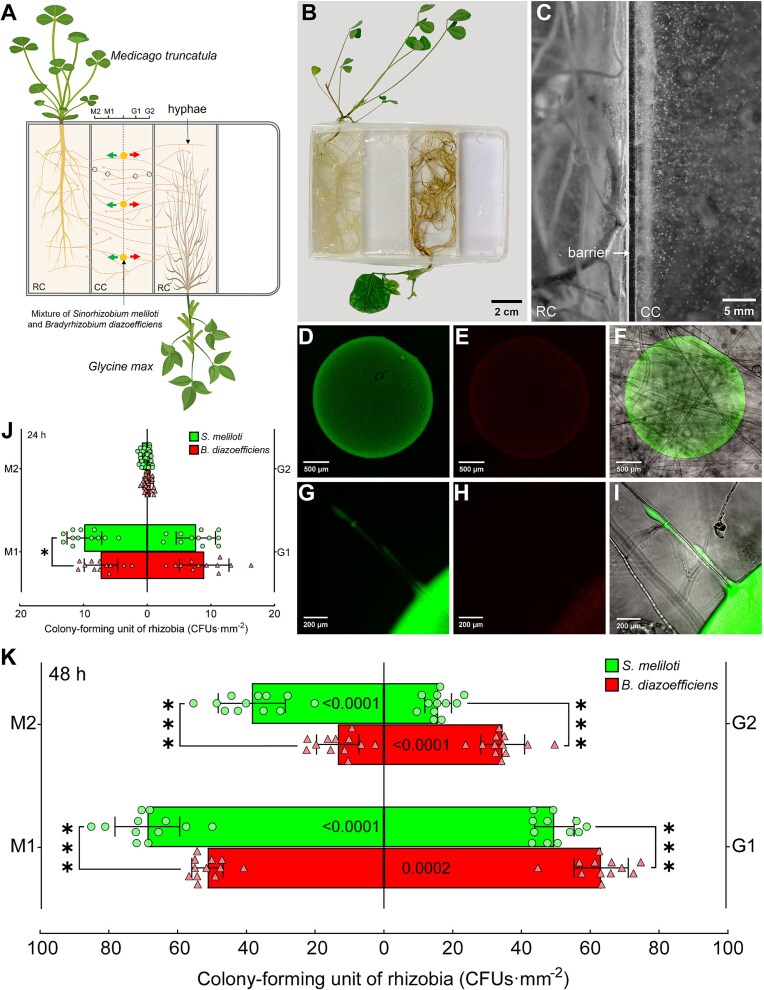
Mycelia-based migration assay of *S. meliloti* and *B. diazoefficiens* in an *in vitro* quadri-compartmented Petri plate system. (A) Schematic representation of the quadri-compartmented *in vitro* culture system. Dots in the HC indicate a mixture of *S. meliloti* and *B. diazoefficiens*. Left-pointing arrows indicate migration towards *M. truncatula*; right-pointing arrows indicate migration towards *G. max*. RC: root compartment; CC: central compartment. Dashed circles indicate the sampling points. Sampling points M1 and M2 are located 0.7 cm and 1.5 cm from the inoculation site toward *M. truncatula*, respectively; G1 and G2 are located 0.7 cm and 1.5 cm toward *G. max*, respectively. (B) Photograph of the quadri-compartmented Petri plate system. (C) Hyphae of *R. irregularis* crossing the barrier from the RC to the CC. (D-F) Fluorescence micrographs of the inoculation site: (D) *S. meliloti*, (E) *B. diazoefficiens*, and (F) merged image of both. (G-I) Fluorescence micrographs of (G) *S. meliloti* and (H) *B. diazoefficiens* migrating along AM fungal hyphae in the CC, and (I) merged image of both. (J, K) Colony-forming units (CFU·mm^−2^) of *S. meliloti* and *B. diazoefficiens* at sampling points M1, M2, G1, and G2 at (J) 24 h and (K) 48 h post-inoculation. Data are presented as means ± SD (*n* = 12 biological replicates). Asterisks indicate significant differences (^*^0.01 ≤ *P* ≤ 0.05; ^***^P ≤ 0.0001) determined by Student’s *t*-test for each sampling point. Numbers on the bar chart represent *P* value from Student’s *t*-test comparing CFU of the same rhizobial species at corresponding sampling points on opposite side of the inoculation site.

**In vitro *experimental design –*** a CMN of *R. irregularis* was connected with *M. truncatula* and *G. max* in a quadri-compartmented Petri plate (Fig. 3A, B). The setup consisted of two side root compartments (RCs), one containing *M. truncatula* and the other *G. max*, both associated with *R. irregularis*. The two RCs (25 ml MSR^min½N^ medium) were separated from a central compartment (CC, 25 ml MSR^min0N^ medium) by plastic barriers. At week 13, three hyphae in the CC were inoculated with 1 μl of mixed *S. meliloti* and *B. diazoefficiens* (9 × 10^5^ CFU·ml^−1^). Bacterial migration was quantified at 24 and 48 h via CFU counts on selective media.

**Figure 4:**
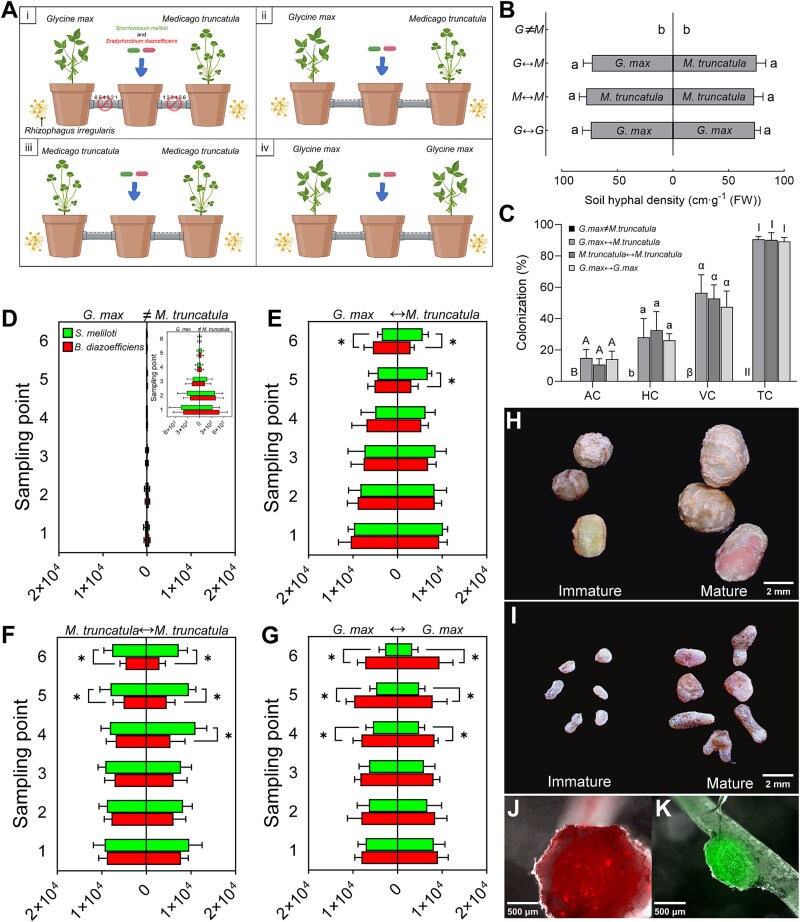
Greenhouse experimental system for studying rhizobial migration and nodulation in *G. max* and *M. truncatula* linked by common mycorrhizal networks. (A) Schematic representation of the greenhouse three-compartment pot system, with numbers (1–6) on the pipes indicating different sampling holes. i: *G. max* and *M. truncatula* in satellite compartments connected to the CC via pipes with 5 μm mesh (*G.max≠M.truncatula*); ii: *G. max* and *M. truncatula* in satellite compartments connected to the CC via pipes with 41 μm mesh (*G.max↔M.truncatula*); iii: *M. truncatula* in both satellite compartments and connected to the CC via pipes with 41 μm mesh (*M.truncatula↔M.truncatula*); iv: *G. max* in both satellite compartments and connected to the CC via pipes with 41 μm mesh (*G.max↔G.max*). (B) Hyphal density in the pipes. Data are presented as means ± SD (*n* = 6 biological replicates). Different lowercase letters (a, b) indicate significant differences between pipes (one-way ANOVA with Tukey’s test, *P* ≤ 0.05). (C) Mycorrhizal colonization of *P. lanceolata* roots in the CC after 4 months of growth. Data are presented as means ± SD (*n* = 6 biological replicates). Different uppercase letters (A, B), lowercase letters (a, b), Greek letters (α, β), and Roman numerals (I, II) indicate significant differences between treatments for arbuscular colonization (AC), hyphal colonization (HC), vesicle colonization (VC), and total colonization (TC), respectively (one-way ANOVA with Tukey’s test, *P* ≤ 0.05). (D-G) Colony-forming units (CFU·g^−1^ fresh substrate) of *S. meliloti* and *B. diazoefficiens* at different sampling points (holes 1–6, with increasing distance from the CC) in the pipes of (D) *G.max≠M.truncatula*, (E) *G.max↔M.truncatula*, (F) *M.truncatula↔M.truncatula*, and (G) *G.max↔G.max* treatments at 9 days post-inoculation (dpi). The inset in Fig. 4D displays the same data as the main panel D but uses an adjusted x-axis scale to better visualize variations between sampling points for this treatment. Data are means ± SD (*n* = 3 biological replicates, with three technical replicates per sample). An asterisk (^*^) indicates *P* ≤ 0.05 (Student’s *t*-test for each sampling hole). (H, I) mature and immature nodules extracted from roots of (H) *G. max* and (I) *M. truncatula*. (J, K) epifluorescence microscopy images of nodules in the roots of mycorrhizal colonized (J) *G. max* and (K) *M. truncatula*.

***Greenhouse experimental design –*** A three-compartment pot system (0.3 L each) connected by perforated pipes was used, with *G. max* and/or *M. truncatula* in satellite compartments and a CMN established via *R. irregularis* (Fig. 4A; Fig. S3). Four treatments were designed, varying in leguminous species combinations (*G. max* and/or *M. truncatula*) and pipe mesh sizes (5 μm or 41 μm, Fig. S4). After CMN stabilization, *S. meliloti* and *B. diazoefficiens* (5 ml, 9 × 10^6^ CFU·ml^−1^) were inoculated in the CC. Migration was assessed over 9 days via CFU counts, and nodulation evaluated at 6 and 8 weeks.

### Data analysis

Prior to statistical analysis, the homogeneity of variance and normality of distribution were assessed using the Levene and Shapiro–Wilk tests, respectively. To attain normality and homoscedasticity of data, all the variables were transformed by taking the base 10 logarithm, except for the root colonization, which were arcsin transformed. Analysis of variance (ANOVA) and student’s *t*-tests were conducted in SAS (8.1) to evaluate the statistical significance of the experimental data. Significance between treatments was determined at 0.05 using the Tukey post-hoc test. Binding affinities between host-specific flavonoids and NodD1 proteins were assessed via *in silico* molecular docking (see Supplementary for details).

All schematics and composite figures in this study were designed using Adobe Illustrator 2023. Some elements, such as plant models and root system models, were sourced from BioRender. Quantitative results, presented as bar charts, were generated using GraphPad Prism (version 10.1.0). The Venn diagram was created using the Venn Diagram plugin in Origin 2022. Principal Component Analysis (PCA) and heatmap visualizations were performed using the Chiplot online portal (https://www.chiplot.online/). No images or figures were created by artificial intelligence (AI) or AI-assisted tools.

## Results

### Root nodulation of *M. truncatula* and *G. max* by *S. meliloti* and *B. diazoefficiens*

To evaluate the host specificity of rhizobial nodulation, a mono-compartmented Petri plate system was established, with *M. truncatula* or *G. max* inoculated with either *S. meliloti* or *B. diazoefficiens*, or PBS as a control (Fig. 1A, B). After 6 weeks of growth, nodules were manually counted and categorized as mature or immature based on their color and morphology.

In the *M.truncatula^S.meliloti^* treatment, 155 ± 8 nodules were counted, including 54 ± 6 mature nodules (35% ± 0.04) and 101 ± 11 immature nodules (65% ± 0.04) (Fig. 1C, E, G). No nodules were detected in the *M.truncatula^B.diazoefficiens^* or *M.truncatula*^PBS^ treatments. For the *G.max^B.diazoefficiens^* treatment, 13 ± 3 nodules were formed, of which 9 ± 3 were mature (70% ± 0.10) and 4 ± 1 were immature (30% ± 0.10) (Fig. 1D, F, G). No nodules were observed in the *G.max^S.meliloti^* or *G.max*^PBS^ treatments.

### Host-specific flavonoid profiles in AM fungal ERM exudates

To investigate the chemical signals mediating host-specific rhizobial recruitment, exudates from the ERM of *R. irregularis* connected to either *M. truncatula* or *G. max* were analyzed using a bi-compartmented Petri plate system (Fig. 2A, B). ERM exudates were collected from the HCs of four treatments (Fig. 2A): RC*^G.max^*/HC^+*R.irregularis*^, RC*^G.max^*/HC^–*R.irregularis*^, RC*^M.truncatula^*/HC^+*R.irregularis*^, and RC*^M.truncatula^*/HC^–*R.irregularis*^. Metabolomic profiling of the four treatments revealed distinct host-specific signatures.

Principal Component Analysis (PCA) showed clear separation of exudates composition based on the plant to which the AM fungus was linked (Fig. 2C). PC1 (65.5% of variation) separated the RC*^G.max^*/HC^+*R.irregularis*^ treatment from the RC*^M.truncatula^*/HC^+*R.irregularis*^ treatment, indicating distinct metabolomic profiles. The treatments without ERM in the HC clustered together and could also be separated from the two treatments with *R. irregularis*.

Of the 28 122 features identified (minimum intensity ≥1000 counts in at least one sample group), the ANOVA (*P* ≤ 0.01) revealed 11 749 significant features. Among 1457 features with MS/MS spectra, 56 were annotated as flavonoids (Supplemental dataset). The Venn diagram (Fig. 2D) highlighted host-specific flavonoid profiles: four flavonoids were exclusive to RC*^G.max^*/HC^+*R.irregularis*^: irilone (isoflavone), erylatissin A (isoflavone derivative), daidzin (isoflavone glycoside), and 6"-O-malonyldaidzin (isoflavone glycoside), suggesting transport from *G. max* roots via AM fungal hyphae. Five features (four kinds of flavonoids) were unique to RC*^M.truncatula^*/HC^+*R.irregularis*^: DL-liquiritigenin (flavanone), naringenin (flavanone), sakuranetin (flavonoid derivative), and 3,7-dimethylquercetin (flavonoid derivative). Additionally, 22 flavonoids were found in both treatments with ERM in the HC, indicating common metabolites transported by AM fungal hyphae. The heatmap confirmed distinct clustering of these flavonoids, supporting host-specific chemical signals transported through AM fungal hyphae (Fig. 2E).

To assess the potential of these host-specific flavonoids to interact with rhizobial NodD1 proteins, *in silico* molecular docking was performed (Fig. 2F). Flavonoids detected exclusively in RC*^G.max^*/HC^+*R.irregularis*^ and RC*^M.truncatula^*/HC^+*R.irregularis*^ treatments were docked with the NodD1 proteins of *B. diazoefficiens* and *S. meliloti*, respectively. Daidzin exhibited the strongest binding affinity to *B. diazoefficiens* NodD1 (−8.3 Kcal·mol^−1^), followed by 6"-O-malonyldaidzin (−8.1 Kcal·mol^−1^), irilone (−7.3 Kcal·mol^−1^), and erylatissin A (−6.9 Kcal·mol^−1^). DL-liquiritigenin showed the highest affinity to *S. meliloti* NodD1 (−7.9 Kcal·mol^−1^), followed by 3,7-dimethylquercetin (−7.6 Kcal·mol^−1^), sakuranetin (−7.5 Kcal·mol^−1^), and naringenin (−7.3 Kcal·mol^−1^).

### CMN enabled rhizobia to migrate preferentially toward their specific hosts *in vitro*

To investigate whether rhizobia exhibit host-specific preferred migration along the CMN, an *in vitro* quadri-compartmented Petri plate system was used, connecting *M. truncatula* and *G. max* through a CC containing a CMN of *R. irregularis* (Fig. 3A-C). Following co-inoculation of *S. meliloti* and *B. diazoefficiens* onto AM fungal hyphae in the CC (Fig. 3D-I), we encountered technical limitations in fluorescence-based quantification due to the stronger signal intensity of GFP-tagged *S. meliloti* (Fig. 3D, G) compared to mCherry-tagged *B. diazoefficiens* (Fig. 3E, H), which resulted in complete spectral overlap in the merged images (Fig. 3F, I). To address this issue, we used selective plating for quantitative comparison of rhizobial migration.

At 24 h post-inoculation, CFU analysis revealed a significant predominance of *S. meliloti* over *B. diazoefficiens* at the M1 sampling point, whereas no significant differences were observed at other sampling points (Fig. 3J). At 48 h, both rhizobia species displayed distinct host-oriented migration patterns via the CMN (Fig. 3K). *S. meliloti* exhibited significantly higher CFUs than *B. diazoefficiens* at both M1 and M2 sampling points. Conversely, *B. diazoefficiens* preferentially migrated toward *G. max*, showing significantly higher CFUs than *S. meliloti* at the G1 and G2 sampling points (Fig. 3K). At their respective sampling distances (0.7 cm for M1 and G1, 1.5 cm for M2 and G2), *S. meliloti* and *B. diazoefficiens* demonstrated significantly higher CFU counts than those migrating toward incompatible hosts (Fig. 3K).

### Greenhouse validation of CMN-mediated host-specific rhizobial recruitment

To validate the role of CMN in the migration and in increasing the concentrations of host-specific rhizobia in the substrate close to the plant host roots, a three-compartment greenhouse pot system was used (Fig. 4A; Fig. S3). This setup linked legumes through pipes with either 41 μm or 5 μm mesh barriers, permitting or restricting fungal hyphal growth, respectively. Four treatments were established: *G.max≠M.truncatula* (Fig. 4A-i), *G.max*↔*M.truncatula* (Fig. 4A-ii), *M.truncatula*↔*M.truncatula* (Fig. 4A-iii), and *G.max*↔*G.max* (Fig. 4A-iv). Hyphal density, rhizobial migration, and nodulation patterns were analyzed to examine CMN-mediated host specificity.

#### Fungal colonization and CMN formation

Fungal colonization was assessed by quantifying hyphal density in the pipes (Fig. 4B) and by evaluating the AM fungal root colonization of *P. lanceolata* in the CC (Fig. 4C). In the *G.max≠M.truncatula* treatment, no hyphae in the pipes and no root colonization of *P. lanceolata* were detected. By contrast, in the other three treatments (*G.max*↔*M.truncatula*, *M.truncatula*↔*M.truncatula*, and *G.max*↔*G.max*), hyphae were observed in the pipes and root colonization detected in *P. lanceolata.* Across these three treatments, no significant differences were observed in hyphal density in the pipes (Fig. 4B) or in any of the measured parameters (i.e. AC, HC, VC, TC) of AM fungal root colonization of *P. lanceolata* (Fig. 4C).

#### Rhizobial migration along CMN

Rhizobial migration was quantified at six sampling points (i.e. holes 1–6) along the pipes at 1, 3, 5, 7 (Fig. S5), and 9 (Fig. 4D-G) dpi. Independent of the dpi, a significantly lower concentration of *S. meliloti* and *B. diazoefficiens* was observed in the *G.max≠M.truncatula* treatment at all sampling points, compared to the other three treatments with AM fungal hyphae present in the pipes. In this treatment, the concentration of rhizobia progressively decreased with distance from the CC, and by day 9, rhizobia were almost undetectable at the sixth sampling point. There were no significant differences between *S. meliloti* and *B. diazoefficiens* at any sampling point in this treatment.

In the *G.max*↔*M.truncatula* treatment, both *S. meliloti* and *B. diazoefficiens* concentrations decreased with increasing distance from the CC at 1, 3, 5, 7, and 9 dpi. During the first 7 days, no significant differences were observed between the concentrations of the two rhizobia at any sampling point. On day 9, at the fifth and sixth sampling points the concentrations of *S. meliloti* were significantly higher than *B. diazoefficiens* in the pipes connected to *M. truncatula*. Conversely, the concentration of *B. diazoefficiens* was significantly higher than *S. meliloti* at the sixth sampling point in the pipes connected to *G. max.*

In the *M.truncatula*↔*M.truncatula* treatment, at 1 and 3 dpi, *B. diazoefficiens* and *S. meliloti* concentrations decreased with distance from the CC. During these early time points, no significant differences were found between the two rhizobia at any sampling point. Starting at 5 dpi, *S. meliloti* concentrations became significantly higher than *B. diazoefficiens* at one side of the sixth sampling point. At 7 dpi, this trend extended to both sides of the sixth sampling point and persisted through 9 dpi, where *S. meliloti* concentrations were significantly higher than *B. diazoefficiens* at the fifth and sixth sampling points. In addition, a similar trend was observed at the fourth sampling point in one of the pipes at 9 dpi.

In the *G.max*↔*G.max* treatment, *B. diazoefficiens* and *S. meliloti* concentrations decreased with distance from the CC at 1 and 3 dpi. During these early time points, no significant differences were observed between the two rhizobia at any sampling point. At 5 and 7 dpi, *B. diazoefficiens* concentrations became significantly higher than *S. meliloti* at one side of the fifth sampling point and both sides of the sixth sampling point. At 9 dpi, this difference extended to both sides of the sixth sampling point, where *B. diazoefficiens* concentrations were significantly higher than *S. meliloti* at the fourth, fifth, and sixth sampling points. In addition, at 9 dpi, *S. meliloti* concentrations exhibited a pronounced decline with distance, particularly at the sixth sampling point, whereas *B. diazoefficiens* showed no such trend.

#### Rhizobial establishment in the substrate close to the roots

**Figure 5:**
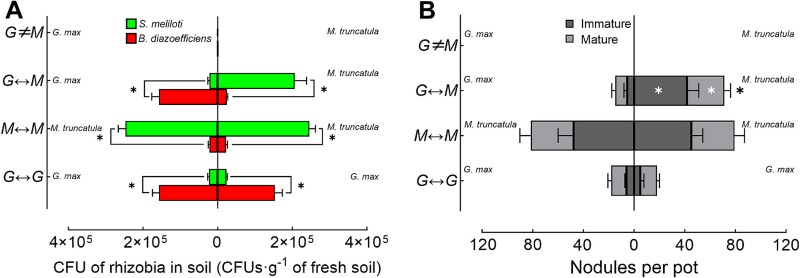
Rhizobial concentrations and nodulation in satellite compartments at 8 weeks post-inoculation (wpi) in greenhouse experiment. (A) Colony-forming units (CFU·g^−1^ fresh substrate) of *S. meliloti* and *B. diazoefficiens* in the satellite compartments in the four treatments at 8 wpi with rhizobia in the CC. Data are means ± SD (*n* = 6 biological replicates, with three technical replicates per sample). An asterisk (^*^) indicates significant difference (*P* ≤ 0.05, Student’s *t*-test) within each satellite compartment. (B) Numbers of immature and mature nodules on roots of *G. max* and *M. truncatula* in the satellite compartments of the four treatments at 8 wpi with rhizobia in the CC. Data are means ± SD (*n* = 6 biological replicates, with three technical replicates per sample). White asterisks (^*^) on the bars for immature nodules (dark gray) and mature nodules (light gray) indicate significant differences between the two satellite compartments within each treatment (*P* ≤ 0.05, Student’s *t*-test). Black asterisks (^*^) adjacent to the bars denote significant differences in total nodule numbers between the two satellite compartments for each treatment (*P* ≤ 0.05, Student’s *t*-test). G *≠ M* (*G.max≠M.truncatula*): *G. max* and *M. truncatula* in satellite compartments connected to the CC via pipes with 5 μm mesh; *G↔M* (*G.Max↔M.Truncatula*): *G. max* and *M. truncatula* in satellite compartments connected to the CC via pipes with 41 μm mesh; *M↔M* (*M.truncatula↔M.truncatula*): Both satellite compartments with *M. truncatula* and connected to the CC via pipes with 41 μm mesh; *G↔G* (*G.max↔G.max*): Both satellite compartments with *G. max* and connected to the CC via pipes with 41 μm mesh.

Rhizobial concentrations in the substrate close to the roots were measured 6 (Fig. S6A) and 8 (Fig. 5A) wpi. At 8 wpi, we found that in the *G.max≠M.truncatula* treatment, rhizobial concentrations in the substrate close to the roots of both *M. truncatula* (222 ± 120 CFU·g^−1^ for *S. meliloti* and 244 ± 124 CFU·g^−1^ for *B. diazoefficiens*) and *G. max* (233 ± 150 CFU·g^−1^ for *S. meliloti* and 200 ± 132 CFU·g^−1^ for *B. diazoefficiens*) were extremely low. In the *G.max↔M.truncatula* treatment, *S. meliloti* concentration (2.1 ± 0.3 × 10^5^ CFU·g^−1^) was significantly higher than *B. diazoefficiens* (0.24 ± 0.03 × 10^5^ CFU·g^−1^) in the rhizosphere of *M. truncatula*. Conversely, in the rhizosphere of *G. max*, *B. diazoefficiens* concentration (1.6 ± 0.2 × 10^5^ CFU·g^−1^) was significantly higher than *S. meliloti* (0.22 ± 0.04 × 10^5^ CFU·g^−1^). In the *M.truncatula↔M.truncatula* treatment, both *M. truncatula* substrate close to the roots were dominated by *S. meliloti* (2.5 ± 0.2 × 10^5^ CFU·g^−1^) and 2.4 ± 0.2 × 10^5^ CFU·g^−1^), respectively), with significantly higher concentrations than *B. diazoefficiens* (0.22 ± 0.03 × 10^5^ CFU·g^−1^ and 0.22 ± 0.04 × 10^5^ CFU·g^−1^, respectively). Similarly, in the *G.max↔G.max* treatment, both *G. max* substrate close to the root were dominated by *B. diazoefficiens* (1.6 ± 0.2 × 10^5^ CFU·g^−1^ and 1.5 ± 0.2 × 10^5^ CFU·g^−1^, respectively), with significantly higher concentrations than *S. meliloti* (0.16 ± 0.02 × 10^5^ CFU·g^−1^ and 0.15 ± 0.02 × 10^5^ CFU·g^−1^, respectively). These statistically significant patterns were consistently observed at 6 wpi across all treatments (Fig. S6A).

#### Host-specific nodulation

Nodulation was analyzed 6 (Fig. S6B) and 8 (Fig. 4H-K; Fig. 5B) wpi. At 8 wpi, no nodules were observed on *G. max* or *M. truncatula* roots in the *G.max≠M.truncatula* treatment. In the *G.max↔M.truncatula* treatment, 15 ± 4 nodules (10 ± 3 mature and 5 ± 3 immature) were formed on the roots of *G. max* and 71 ± 10 (29 ± 5 mature and 42 ± 9 immature) on the roots of *M. truncatula*. In the *G.max↔G.max* treatment, both plants formed 18 (± 3 and ± 4, respectively) nodules each, with one plant producing 12 ± 3 mature and 6 ± 1 immature nodules, and the other producing 13 ± 2 mature and 5 ± 3 immature nodules. In the *M.truncatula↔M.truncatula* treatment, the two *M. truncatula* plants formed 82 ± 17 and 79 ± 10 nodules, respectively. The nodules included 34 ± 9 and 34 ± 8 mature nodules, and 48 ± 12 and 45 ± 9 immature nodules. Similar trends were observed at 6 wpi, with comparable patterns in nodule formation and distribution across all treatments, and these differences were statistically significant (Fig. S6B).

## Discussion

This study examined the central role of the CMN of the AM fungus *R. irregularis* in mediating the migration of *S. meliloti* and *B. diazoefficiens*, and subsequent nodulation in their respective legume hosts, *M. truncatula* and *G. max*. By combining *in vitro* and greenhouse experiments with metabolomic analyses, we have revealed the dual function of this CMN: it acts both as a fungal highway facilitating the dispersal of rhizobia and as a carrier of chemical signals (i.e. flavonoids) guiding the migration of rhizobia towards their specific host. This selective tropism transforms the CMN into a sophisticated “navigation system”, which orient the rhizobia through the complex soil matrix with great precision.

In our study, the high specificity of rhizobia-legume interaction was confirmed by the exclusive nodulation of *S. meliloti* with the roots of *M. truncatula* and of *B. diazoefficiens* with the roots of *G. max*. This host-specific nodulation reflects the tightly regulated relationships between rhizobia and their legume partners, where recognition signals are fine-tuned to ensure compatibility [31, 32]. Such specificity is likely mediated by the exchange of signal molecules, including flavonoids from the plant to the rhizobia and Nod factors from the rhizobia to the plant, a process well-documented as critical to initiate symbiosis [33]. Our results further indicated that this specific recognition mechanism may also be mediated by the hyphae of AM fungi. These belowground conduits transport and release compounds, such as flavonoids, specific to the legume to which they are linked, acting as chemoattractants for rhizobia towards their compatible host.

Central to our study was the demonstration that the CMN formed between two different legume species by *R. irregularis* served as a physical conduit, or “fungal highway”, for the migration of rhizobia towards their specific host. This concept of “fungal highway”, as presented in previous studies [34, 35], indicates that hyphal surfaces, coated with water films, create microenvironments favorable to bacterial motility, particularly in soils where diffusion alone can limit microbial propagation. Using an *in vitro* compartmented system, we observed that *S. meliloti* and *B. diazoefficiens* migrated along the AM fungal hyphae with a higher presence towards *M. truncatula* for the first and towards *G. max* for the second. Interpreting the CFU data from our *in vitro* system (Fig. 3) requires consideration of whether differences arise from differential migration or potential differential growth at the sampling sites. Although some localized bacterial growth within the central compartment medium over 24–48 h cannot be entirely discounted, several factors suggest that active, host-preference-driven migration along AM fungal hyphae is the predominant mechanism. These include the nutrient-limited nature of the hyphal support medium, the clear host-specific directional accumulation of rhizobia, and the visual evidence of bacterial association with fungal hyphae. Thus, we interpret the observed CFU differences as primarily indicative of differential bacterial migration efficiency and/or preference. This specific migration suggests that the CMN not only provides a facilitated pathway for rhizobial dispersal to new niches but also helps the bacteria to move preferentially towards their compatible hosts, at a reduced energetic cost. This fungal path was suggested by recent studies of improved migration efficiency due to facilitated flow velocity and chemical guidance along fungal hyphae, further contextualized within the framework of optimized symbiotic interactions and resource allocation [10, 36–38]. The greenhouse experiment using a compartmented pot system further corroborated this mechanism under more natural conditions (Fig. 4). In the treatment with CMN connecting the two legumes species, rhizobial concentrations decreased progressively with distance from the inoculation point towards the compatible host, indicating a hyphal-mediated transport. In contrast, in the treatment without CMN connecting plants, only a very limited migration was observed, underlining the key role of CMNs in bridging spatial gaps between rhizobia and their hosts. The analysis of CFU of both rhizobia revealed dynamic patterns: in the treatment linking the two different legumes by the CMN, we observed no significant differences in the CFUs of *S. meliloti* and *B. diazoefficiens* during the first 7 dpi at any sampling point in the pipes (Fig. S5). This suggests that, initially, the migration of both rhizobia along the hyphae may be non-directional, with no clear preference for either host. Conversely 9 dpi, a significant difference was observed (Fig. 4E). Close to *M. truncatula*, the concentration *S. meliloti* was significantly higher than that of *B. diazoefficiens*, whereas the reverse was observed near *G. max*. This difference indicates that migration becomes increasingly oriented as rhizobia approach their specific hosts, likely due to the increasing concentration of specific flavonoids.

In addition to physical transport, our study revealed an important role for the hyphae of CMN in transporting and releasing signal molecules from the host to which they are connected, thereby orienting the migration of rhizobia towards their specific hosts. The metabolomic profiling of ERM exudates (in particular the flavonoids) collected in the hyphal compartment of the bi-compartmented *in vitro* culture system differed between plants to which the hyphae are connected (Fig. 2A, B). The perception of these flavonoids by rhizobia is mediated by NodD proteins, transcriptional regulators of the LysR family that undergo a conformational change upon binding specific flavonoids, enabling them to activate nod genes by binding to conserved DNA sequences known as nod boxes [39, 40]. This specificity, driven by unique flavonoid cocktails from each legume and varying NodD isoforms in rhizobia, is a key determinant of symbiotic host range [41].

For the exudates of ERM connected to *G. max*, isoflavones including daidzin, 6"-O-malonyldaidzin, irilone and erylatissin A were detected (Fig. 2F), with binding affinities to *B. diazoefficiens* NodD1 of −8.3 Kcal·mol^−1^, −8.1 Kcal·mol^−1^, −7.3 Kcal·mol^−1^ and − 6.9 Kcal·mol^−1,^ respectively. Daidzin, a glycosylated form of daidzein, stands out as a powerful inducer of nod genes, as previously indicated [40, 42]. Its high affinity for NodD1 underlines its central role in the initiation of symbiosis by triggering transcriptional activation of nodulation genes. Similarly, 6"-O-malonyldaidzin not only contributes to *nod* gene induction but also appears to modulate the secretion of other isoflavones [43], suggesting a regulatory function that fine-tunes the symbiotic dialogue between *G. max* and *B. diazoefficiens*. Irilone, although more commonly associated with red clover [44], shows moderate NodD1 affinity in our study, suggesting a possible secondary role in soybean, perhaps as a chemoattractant attracting *B. diazoefficiens* to the host or as a modulator of wider microbial interactions in the rhizosphere. Erylatissin A, had the lowest affinity among the four compounds above. This compound lacks extensive studies in soybean symbiosis. Its presence in ERM exudates suggests that it might contribute to a broader chemical signature, potentially enhancing *B. diazoefficiens* recognition or subtly influencing rhizosphere ecology.

For the exudates of ERM connected to *M. truncatula*, the most abundant compounds were flavanones like DL-liquiritigenin and naringenin, and alongside flavonoid derivatives like sakuranetin and 3,7-dimethylquercetin, with binding affinities to *S. meliloti* NodD1 of −7.9 Kcal·mol^−1^, −7.3 Kcal·mol^−1^, −7.5 Kcal·mol^−1^ and − 7.6 Kcal·mol^−1^, respectively (Fig. 2F). DL-liquiritigenin appears to be a key player, not only because of its high NodD1 binding affinity, but also because of its demonstrated ability to rescue nodulation in flavonoid-deficient *M. truncatula* mutants [45]. This ability indicates that it can either induce nodulation genes or act synergistically with other signals to establish symbiosis. Naringenin, a precursor in flavonoid biosynthesis, can stimulate the binding of *S. meliloti* NodD1 to *nod* gene promoters, although it does not induce gene expression [46]. It has also been shown to act as a competitive inhibitor of known *nod* gene inducers like luteolin [39], which suggests a regulatory role in fine-tuning *S. meliloti* responses. Sakuranetin, although less studied in this specific symbiosis, is known as a phytoalexin in other plant species [47]. It could integrate symbiotic signaling with defense responses, with AM fungal hyphae potentially transporting it to amplify *M. truncatula*’s chemical signature and aid *S. meliloti* in distinguishing its host from competitors (*B. diazoefficiens* in this study) or pathogens [48]. Similarly, for 3,7-dimethylquercetin, there is a lack of studies on this compound, but its methylated structure, a modification highlighted in *M. truncatula* flavonoids [49], could reinforce host specificity, possibly by stabilizing NodD1 interactions or modulating rhizosphere microbial interactions. These compounds were absent from the exudates of the hyphal-free controls, highlighting the active participation of AM fungi in the transport and release of signals.

By transporting and releasing these host-specific signals along a probable root-to-hyphal tip concentration gradient, AM fungi play a crucial role in guiding the rhizobia towards their appropriate host. We hypothesize that in the central region of the CMN (i.e. at equal distance of the two legumes), the signals released by the hyphae linking *M. truncatula* and *G. max* could not be differentiated, resulting in the absence of a clear gradient towards either host [50, 51]. In this central zone, rhizobia can move randomly in both directions, with no clear preference for one host or the other. As they migrated closer to one legume or another, they probably perceived greater gradients of host-specific flavonoids, triggering chemotaxis towards their compatible host. This hypothesis is supported by previous studies [6, 52], who demonstrated that bacteria can detect and respond to chemical gradients over time, resulting in chemotaxis towards specific attractants. This phenomenon can be amplified by the coenocytic nature of AM fungal hyphae facilitating signal diffusion across the inoculation point, with flavonoids from one plant potentially present on the opposite side, complicating initial directional cues until a significant dominant gradient emerges near the host [53, 54].

The ability of CMNs to transport and release specific signals from the plant to whom they are connected resembles a sophisticated “navigation system” within the soil, transmitting host-specific cues that serve as “signposts”. These cues guide *S. meliloti* and *B. diazoefficiens* through the complex “labyrinth” of the soil matrix, directing them precisely to their respective host plants, *M. truncatula* and *G. max.* This resulted in a relatively higher concentration of *S. meliloti* close to *M. truncatula* roots versus *B. diazoefficiens* close to *G. max.*

The high specificity of flavonoid release suggests a co-evolutionary adaptation between AM fungi, legumes, and rhizobia. By transporting and releasing exudates to recruit compatible rhizobia, AM fungi optimize symbiotic outcomes, aligning with a resource exchange framework where they act as intermediaries maximizing nitrogen fixation and nutrient uptake [55]. This role parallels their transport of phosphate-solubilizing bacteria to nutrient-rich patches, highlighting their mediation in plant-microbe interactions [56].

## Conclusion

**Figure 6:**
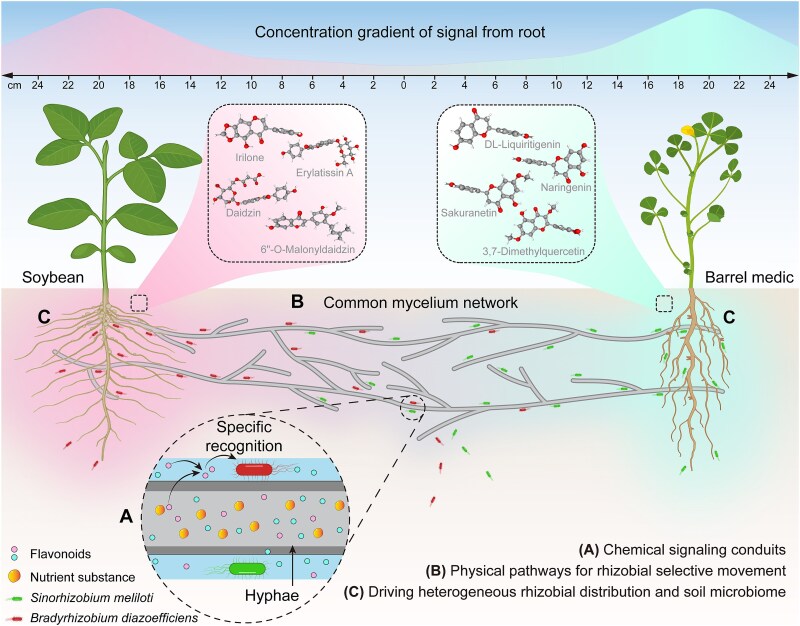
Schematic representation of how the common mycorrhizal network (CMN) of *R. irregularis* guides the selective recruitment of rhizobia to their compatible legume hosts. The CMN connecting soybean (*Glycine max*, left) and barrel medic (*Medicago truncatula*, right) serves as both a fungal highway and a chemical signaling conduit. Host-specific flavonoids – depicted with distinct visual markers associated with soybean on the left (including daidzin, 6"-O-malonyldaidzin, irilone, and erylatissin A) and with distinct visual markers associated with barrel medic on the right (including DL-liquiritigenin, naringenin, sakuranetin, and 3,7-dimethylquercetin) –produced by the roots of legumes are transported and released by the fungal hyphae. These flavonoids establish signal gradients that act as molecular navigation cues for rhizobia. The magnified inset (A) reveals the detailed mechanism of rhizobial migration, showing how *B. diazoefficiens* and *S. meliloti* move along hyphal surfaces in response to these flavonoid signals, which are transported within the hyphal cytoplasm and/or through the external water film. This chemical signaling drives the preferential movement of rhizobia toward their compatible hosts: *B. diazoefficiens* navigates toward soybean, whereas *S. meliloti* is guided to barrel medic, with sharpening gradients of host-specific flavonoids refining their navigation precision as they approach their targets, enhancing nodulation efficiency and nitrogen fixation potential. This sophisticated biological navigation system illustrates how the CMN spatially organizes rhizobial communities in soil, creating heterogeneous microbial distributions that optimize symbiotic partnerships and potentially reshape the broader soil microbiome. The precision of this recruitment mechanism reflects the co-evolutionary adaptations among legumes, rhizobia, and arbuscular mycorrhizal fungi, providing an ecological advantage in nutrient acquisition and highlighting the remarkable complexity of belowground plant-microbe interactions that support sustainable nitrogen cycling in terrestrial ecosystems.

Legume-rhizobia symbiosis typically relies on short-distance root signals, with long-distance dispersal attributed to external vectors. Our *in vitro* and greenhouse experiments show that *R. irregularis* hyphae, via CMN, fill this gap, directing *S. meliloti* and *B. diazoefficiens* to *M. truncatula* and *G. max*, respectively. These networks integrate physical transport with host-specific flavonoid signals, acting as a sophisticated navigation system that ensures precise rhizobial delivery and selective nodulation. Beyond nutrient exchange, CMN spatially organize rhizobia, enhancing symbiotic efficiency and demonstrating an ecological role for AM fungi as regulators of rhizobia distribution in soil. Given their omnipresence in soils, this mechanism improves rhizobial access to compatible hosts, supporting the co-application of AM fungi and rhizobia for sustainable nitrogen fixation. Future studies under fields across diverse legume systems are essential to translate these observations into agricultural practice. This research repositions AM fungi as key mediators of belowground microbial interactions, paving the way for eco-friendly farming strategies (Fig. 6).

## Supplementary Material

Supplementary_information_wraf100

Supplemental_dataset_wraf100

## Data Availability

The authors declare that materials described in the manuscript, including all relevant raw data, will be freely available to any researcher wishing to use them for non-commercial purposes.
