# Supplementary material for: Legume-specific recruitment of rhizobia by hyphae of arbuscular mycorrhizal fungi
Source: ISME J. 2025 May 21;19(1):wraf100. doi: 10.1093/ismejo/wraf100 (PMC12145873; doi:10.1093/ismejo/wraf100)
Supplement: Supplementary_information_wraf100 [file supplementary_information_wraf100.docx]

**Supplementary Information**

[1. Materials and methods 2](#_Toc195172388)

[1.1. Composition of the different Modified-Strullu-Romand (MSR) media 2](#_Toc195172389)

[1.2. Biological material 3](#_Toc195172390)

[1.3. Growth and nodulation of *S. meliloti* and *B. diazoefficiens* on *M. truncatula* and *G. max* 4](#_Toc195172391)

[1.4. *In vitro* experimental design for analyzing flavonoids released by the ERM of *R. irregularis* connected to *G. max* or *M. truncatula* 5](#_Toc195172392)

[1.5. *In vitro* and greenhouse experimental designs with legumes linked by a CMN for mycelia-based migration assay of *S. meliloti* and *B. diazoefficiens* 7](#_Toc195172393)

[1.6. *In silico* binding studies 11](#_Toc195172394)

[2. Results 13](#_Toc195172395)

[3. References 15](#_Toc195172396)

# 1. Materials and methods

## 1.1. Composition of the different Modified-Strullu-Romand (MSR) media

The culture media used were all deriving from the MSR medium [1]: (1) The MSR^min^ medium, without sucrose and vitamins, (2) the MSR^min0N^ with 0 mM N (KNO_3_ and Ca(NO_3_)_2_·4H_2_O were replaced by 75.2 mM KCl and 152 mM CaCl_2_·2H_2_O, respectively) and (3) the MSR^min½N^ containing half the N concentration (1.99 mM) of the normal MSR^min^ medium (i.e., 37.6 mM of KNO_3_ and 76 mM of Ca(NO_3_)_2_·4H_2_O) (Table S1).

Table S1. Concentration of stock solutions (g·L^–1^) to prepare the MSR, MSR^min^, MSR^min½N^, and MSR^min0N^ media

|  |  | **MSR** | **MSR^min^** | **MSR^min½N^** | **MSR^min0N^** |
| --- | --- | --- | --- | --- | --- |
| **Macro-elements** |  |  |  |  |  |
|  | KNO_3_ | 7.6 | 7.6 | 3.8 | - |
|  | KCl | 6.5 | 6.5 | 6.05 | 12.1 |
|  | KH_2_PO_4_ | 0.41 | 0.41 | 0.41 | 0.41 |
|  | MgSO_4_·7H_2_O | 73.9 | 73.9 | 73.9 | 73.9 |
| **Calcium Nitrate** |  |  |  |  |  |
|  | Ca(NO_3_)_2_·4H_2_O | 35.9 | 35.9 | 17.95 | - |
|  | CaCl_2_ | - | - | 8.435 | 16.87 |
| **NaFeEDTA** |  | 1.6 | 1.6 | 1.6 | 1.6 |
| **Micro-elements** |  |  |  |  |  |
|  | MnSO_4_·4H_2_O | 2.45 | 2.45 | 2.45 | 2.45 |
|  | ZnSO_4_·7H_2_O | 0.28 | 0.28 | 0.28 | 0.28 |
|  | H_3_BO_3_ | 1.85 | 1.85 | 1.85 | 1.85 |
|  | CuSO_4_·5H_2_O | 0.22 | 0.22 | 0.22 | 0.22 |
|  | (NH_4_)_6_Mo_7_O_24_·4H_2_O | 0.034 | 0.034 | 0.017 | - |
|  | Na_2_MoO_4_·2H_2_O | 0.0024 | 0.0024 | 0.0257 | 0.049 |
| **Vitamins** |  |  |  |  |  |
|  | Ca panthotenate | 0.18 | - | - | - |
|  | Biotine | 0.18×10^-3^ | - | - | - |
|  | Pyridoxine | 0.18 | - | - | - |
|  | Thiamine | 0.2 | - | - | - |
|  | Cyanocobalamine | 0.08 | - | - | - |
|  | Nicotinic acid | 0.2 | - | - | - |

## 1.2. Biological material

***Rhizophagus irregularis* MUCL 41833**: The arbuscular mycorrhizal (AM) fungus *R. irregularis* (Blaszk., Wubet, Renker & Buscot) C. Walker & A. Schüßler comb. nov. MUCL 41833 was obtained from the Glomeromycota *in vitro* collection (GINCO, Belgium). It was maintained *in vitro* using Ri T-DNA transformed roots of carrot (*Daucus carota* L., clone G3) in bi-compartmented Petri plates (90 mm diameter, VWR International, Belgium) (Fig. S1). The root compartment (RC) contained 25 mL of MSR medium [1], adjusted to pH 5.5, solidified with 3 g·L^–1^ Phytagel (Sigma-Aldrich Co.), and sterilized at 121°C for 15 min under 0.11 MPa pressure in an autoclave. The hyphal compartment (HC) contained 25 mL of MSR^min^ medium. Plates were incubated in the dark at 27°C for 8-12 weeks until extensive extraradical mycelium (ERM) growth was observed in the HC. Roots crossing the plastic barrier separating the RC from the HC were trimmed weekly using sterilized forceps.


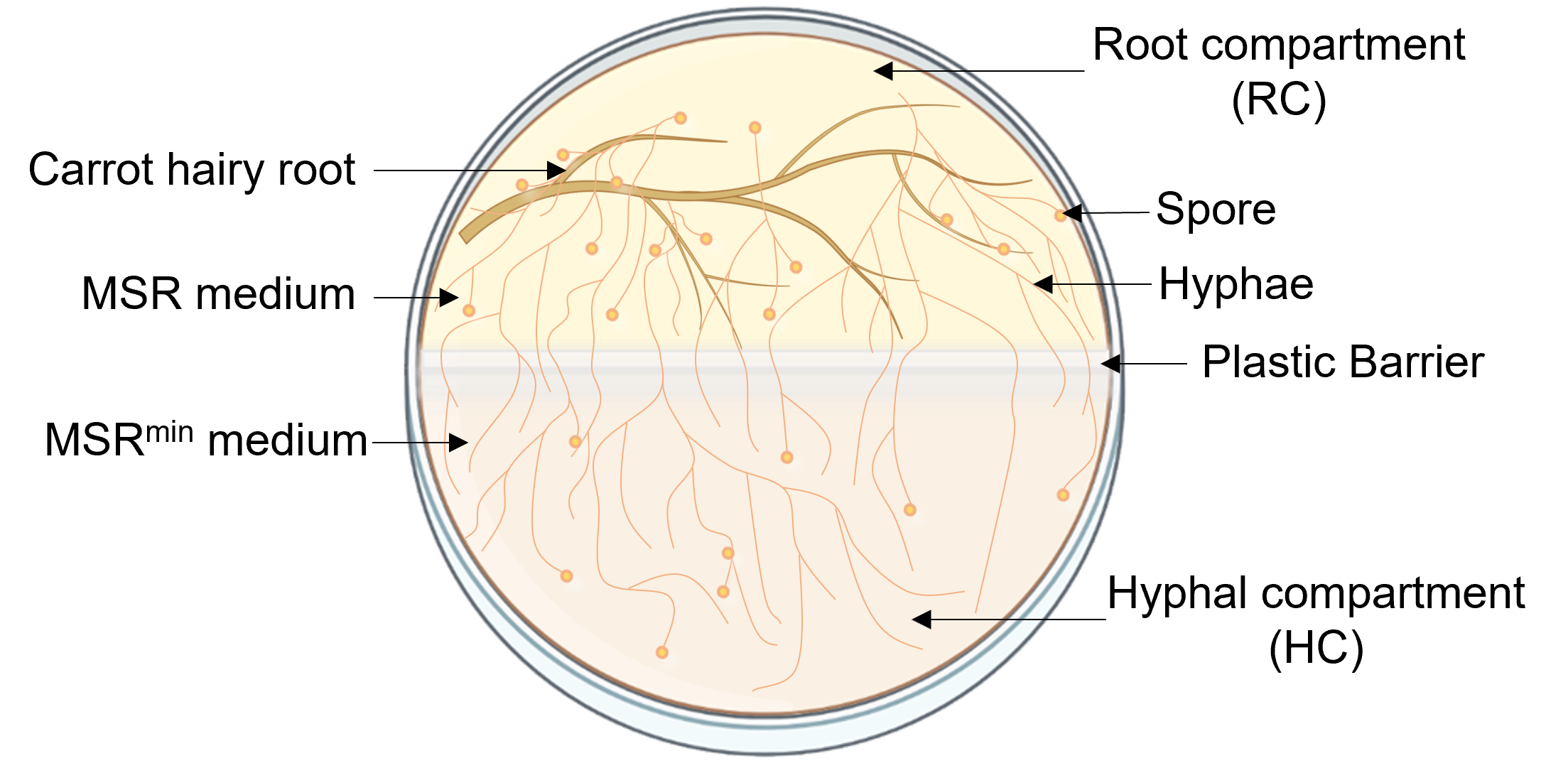


Figure S1. Schematic representation of the root-organ culture (ROC) system.

***Sinorhizobium meliloti* 2011 pHC60-GFP** [2]: This strain, tagged with green fluorescent protein (GFP) and resistant to tetracycline, was provided by the VIB-UGent Center for Plant Systems Biology (Ghent University, Belgium). The methodology for GFP tagging and use in visualizing symbiotic interactions was adapted from previously described approaches [2]. In this strain, GFP is constitutively expressed from the plasmid pHC60, ensuring stable fluorescence for tracking during plant interactions. It was cultured in yeast extract broth (YEB) medium containing 5 g·L^–1^ peptone, 5 g·L^–1^ beef extract, 5 g·L^–1^ sucrose, 1 g·L^–1^ yeast extract, and 0.4 g·L^–1^ MgSO_4_·7H_2_O, adjusted to pH 7.5 and autoclaved at 121°C for 15 min. Tetracycline was added post-autoclaving at 5 µg·mL^–1^ from a stock solution (5 mg·mL^–1^ in 50% ethanol, filter-sterilized with a 0.2 µm Acrodisc Syringe Filter, Pall Corporation, New York, USA). The bacterium was grown at 28°C with shaking at 180 rpm until reaching OD_600_ = 0.8-1.0. For inoculation, cultures were centrifuged at 4000 × g for 10 min, resuspended in sterile phosphate-buffered saline (PBS: 137 mM NaCl, 2.7 mM KCl, 10 mM Na_2_HPO_4_, 1.8 mM KH2PO4, pH 7.4) autoclaved at 121°C for 15 min, and adjusted to 9 × 10^5^ CFU·mL^–1^ based on OD_600_ calibration [3].

***Bradyrhizobium diazoefficiens* mChe-1** [4]: This strain tagged with mCherry (red fluorescent protein) and resistant to spectinomycin was provided by the VIB-UGent Center for Plant Systems Biology (Ghent University, Belgium). Constitutive expression of mCherry was achieved using stable tagging methods, including plasmid-based expression and chromosomal integration, as previously described [4]. It was cultured in YEB medium supplemented with 50 µg·mL^–1^ spectinomycin, prepared as a 50 mg·L^–1^ stock in deionized water, filter-sterilized, and added post-autoclaving. Growth conditions were identical to *S. meliloti*: 28°C, 180 rpm, until OD_600_ = 0.8-1.0. Cultures were harvested and diluted in PBS to 9 × 10^5^ CFU·mL^–1^ as described above.

**Plant Seeds**: Seeds of *Medicago truncatula* L. cv. Jemalong A17 (*M. truncatula*) were supplied by the South Australian Research and Development Institute (SARDI, Australia), *Glycine max* (L.) Merr. (*G. max*) by the VIB-UGent Center for Plant Systems Biology, and *Plantago lanceolata* L. (*P. lanceolata*) by ECOSEM (Corroy-le-Grand, Belgium). Seeds were surface-disinfected in a laminar flow hood by immersion in 8% active chloride bleach (household bleach diluted with deionized water) for 15 min with gentle agitation, followed by three 5-min washes with sterilized deionized water (autoclaved at 121°C for 15 min). Germination occurred in mono-compartmented Petri plates (90 mm diameter) containing 40 mL of MSR medium, incubated at 20°C in the dark for 7 days in an incubator (WTB Binder-78532 Tuttlingen, Germany).

## 1.3. Growth and nodulation of *S. meliloti* and *B. diazoefficiens* on *M. truncatula* and *G. max*

**Setup**: Seven-day-old *G. max* or *M. truncatula* seedlings were transferred to mono-compartmented Petri plates (90 mm diameter). Briefly, the roots were plated on the surface of MSR^min½N^ solidified with 3 g·L^–1^ Phytagel. The shoot extended outside the Petri plate via a 4 mm hole, which was drilled on the plate side, sealed with autoclaved silicone grease (VWR International, Belgium), while the roots remained inside the Petri plate. Plates were sealed with Parafilm (Pechiney, Chicago, IL, USA) and incubated in a growth chamber at 22/18°C (day/night), 70% relative humidity, 16 h·day^-1^ photoperiod, and 225 µmol·m^–2^·s^–1^ photosynthetic photon flux.

**Treatments**: Six treatments were considered: (1 and 2) *G. max* or *M. truncatula* inoculated with a phosphate buffered saline (PBS) solution (*G.max*^PBS^ and *M.truncatula*^PBS^, respectively), (3 and 4) *G. max* or *M. truncatula* inoculated with *S. meliloti* (*G.max^S.meliloti^* and *M.truncatula^S.meliloti^*, respectively), and (5 and 6) *G. max* or *M. truncatula* inoculated with *B. diazoefficiens* (*G.max^B.diazoefficiens^* and *M.truncatula^B.diazoefficiens^*, respectively). The bacterial inoculum consisted of a suspension of 150 µL of the bacteria in PBS solution, at a concentration adjusted to 9 × 10^5^ CFU·mL^–1^ (OD_600_ = 0.1). The bacterial suspension was directly pipetted onto the roots of the plants to ensure direct contact between the bacteria and the root surface. Control treatments received an equivalent volume of PBS without bacteria. Six replicates were considered per treatment.

**Nodule assessment**: After 6 weeks, plants were removed from the Petri plates using sterile forceps, rinsed with deionized water, and blotted dry with sterile paper towels. Roots were examined under a stereomicroscope (Olympus SZ61) at 0.67× magnification with a cold light source. Nodules were counted visually and classified based on color and shape. Mature nodules were identified by their pink to red color indicating active N_2_ fixation, whereas immature nodules were white, meaning that N_2_ fixation had not yet begun [5]. Additionally, nodules were categorized as either determinate or indeterminate based on their shape. Determinate nodules are typically spherical, whereas indeterminate nodules exhibit an elongated shape due to continuous meristematic activity [6].

## 1.4. *In vitro* experimental design for analyzing flavonoids released by the ERM of *R. irregularis* connected to *G. max* or *M. truncatula*

**Setup**: Bi-compartmented Petri plates (90 mm diameter) were prepared with 25 mL MSR^min½N^ medium in the RC and 10 mL liquid MSR^min0N^ medium in the HC. *R. irregularis* was inoculated on the roots in the RC, and the ERM was allowed to grow into the HC. Four treatments were considered: (1) RC*^G.max^*/HC^+^*^R.irregularis^*, (2) RC*^G.max^*/HC^–^*^R.irregularis^*, (3) RC*^M.truncatula^*/HC^+^*^R.irregularis^*, and (4) RC*^M.truncatula^*/HC^–^*^R.irregularis^*. The Petri plates were transferred to a growth chamber (conditions as above) for 13 weeks until numerous hyphae were observed in the HC, confirmed by stereomicroscope observation.

**Medium replacement and collection**: At week 13, the HC liquid medium was aspirated with a sterile pipette under a laminar flow hood, and 10 mL fresh liquid MSR^min0N^ (prepared fresh, autoclaved, and cooled to 25°C) was added. After one week (week 14), the medium was collected into 15 mL centrifuge tubes (Falcon, Corning, USA), snap-frozen in liquid nitrogen, and stored at –80°C for subsequent flavonoid analysis.

**Flavonoids analysis**: Six mL of MSR^min0N^ medium collected in each HC was extracted with 6 mL methanol at room temperature for 1 h, followed by evaporation to dryness under vacuum conditions using a rotary evaporator (Büchi Rotavapor R-100, Switzerland). The dry methanol extracts were resolubilized in 100 µL cyclohexane and 100 µL Milli-Q water, vortexed thoroughly, and then centrifuged at 14000 rpm. Eighty µL of the aqueous phase was filtered using a 96-well filter plate (Millipore MultiScreen, USA). Samples were subjected to Ultra Performance Liquid Chromatography High Resolution Mass Spectrometry (UPLC-HRMS) at the VIB Metabolomics Core Ghent (VIB-MCG). Ten µL of the sample was injected on a Waters Acquity UHPLC (Waters) device connected to a Synapt XS high-definition mass spectrometer (Waters). Chromatographic separation was carried out on an ACQUITY UPLC BEH C18 (150 × 2.1 mm; 1.7 μm) column (Waters) with the column temperature maintained at 40°C. A gradient of two buffers was used for separation: buffer A (water + 0.1% formic acid, pH 3) and buffer B (acetonitrile + 0.1% formic acid, pH 3). Buffer A was decreased from 99% to 50% in 30 min, decreased to 30% in 30 to 35 min, and further decreased to 0% in 35 to 37 min. The flow rate was set to 0.35 mL·min^–1^. Electrospray Ionization (ESI) was applied, and the LockSpray ion source was operated in negative ionization mode under the following conditions: capillary voltage, 3 kV; reference capillary voltage, 2.5 kV; source temperature, 120°C; desolvation gas temperature, 550°C; desolvation gas flow, 800 L·h^–1^; cone gas flow, 50 L·h^–1^. The collision energy for full MS scan was set at 4 eV. For data-dependent acquisition–tandem mass spectrometry (DDA-MS/MS), the low mass ramp was ramped between 6–20 eV, and the high mass ramp was ramped between 20-70 eV. The mass range was set from 50 to 1500 Da, and scan time was set at 0.3 s. Nitrogen (greater than 99.5%) was employed as desolvation and cone gas. Leucine-enkephalin (100 pg·µL^–1^ solubilized in water:acetonitrile 1:1 [v/v], with 0.1% formic acid) was used for lock mass calibration, scanning every 0.75 min at a scan time of 0.3 s. Profile data was recorded through Masslynx (Waters). Data processing was performed with Progenesis QI software version 3.0 (Waters) for chromatogram alignment and compound ion detection. The detection limit was set at medium sensitivity with a minimum peak width of 0.04 min. The data was normalized to all compound ions. The following filters were applied to analyze the data: a minimum ion intensity of 1000 counts in at least one sample group (resulting in 28122 features) and an ANOVA (*P* ≤ 0.01, resulting in 11,749 features). Statistical analyses were performed on ArcSinh-transformed and Pareto-scaled ion intensities. Structural annotation of the significant features was attempted using MS-FINDER (*in silico* fragmentation) [7]. The following parameter settings were applied: formula prediction and structural elucidation by *in silico* fragmenter using all available local databases, cut-off score for structural elucidation: 5, MS1 mass tolerance: 5 ppm, MS2 mass tolerance: 10 ppm, relative abundance cut-off: 0.1%, LEWIS and SENIOR check: TRUE, element ratio check: common range (99.7%), element selection: C, H, N, tree depth: 2.

## 1.5. *In vitro* and greenhouse experimental designs with legumes linked by a CMN for mycelia-based migration assay of *S. meliloti* and *B. diazoefficiens*

The experimental design was adapted from our previous study [3], with modifications to accommodate the two plant species and two bacterial strains.

***In vitro* experimental design**

A quadri-compartmented Petri plate (12.4 × 8.5 cm) featured two side root compartments (RCs) with 25 mL MSRmin½N^min½N^ medium each, containing *M. truncatula* and *G. max* associated with *R. irregularis*, and a central compartment (CC) with 25 mL MSR^min0N^ medium. The Petri plates were incubated as above for 13 weeks until a common mycorrhizal network (CMN) was formed in the CC, confirmed by stereomicroscope observation of hyphal bridges. Three hyphae per plate, visibly connecting the two plants and growing on the CC surface, were inoculated with 1 µL of a 1:1 mixture of *S. meliloti* and *B. diazoefficiens* (0.5 µL each, 9 × 10^5^ CFU·mL^–1^). Bacterial migration was quantified at 24 and 48 h post-inoculation by sampling medium with a 2 mm diameter cork borer at 0.7 cm and 1.4 cm from both sides of the inoculation site (Fig. S2). The medium samples were transferred to 1.5 mL sterile centrifuge tubes containing 0.2 mL of sterile PBS, vortexed for 5 s at 1200 rpm using an MS1 shaker (IKA, Germany), and 20 μL of the suspension was spread onto each selective agar plates using an L-Shape Cell Spreader (VWR). The number of colony forming units (CFUs) of *S. meliloti* was determined on YEB solid medium (15 g·L^–1^ agar) supplemented with 5 μg·mL^–1^ tetracycline, while for *B. diazoefficiens*, it was determined on YEB solid medium (15 g·L^–1^ agar) supplemented with 50 μg·mL^–1^ spectinomycin. The Petri plates were incubated at 28°C for 48 h, and CFUs were counted. Three biological replicates were prepared for each sample.


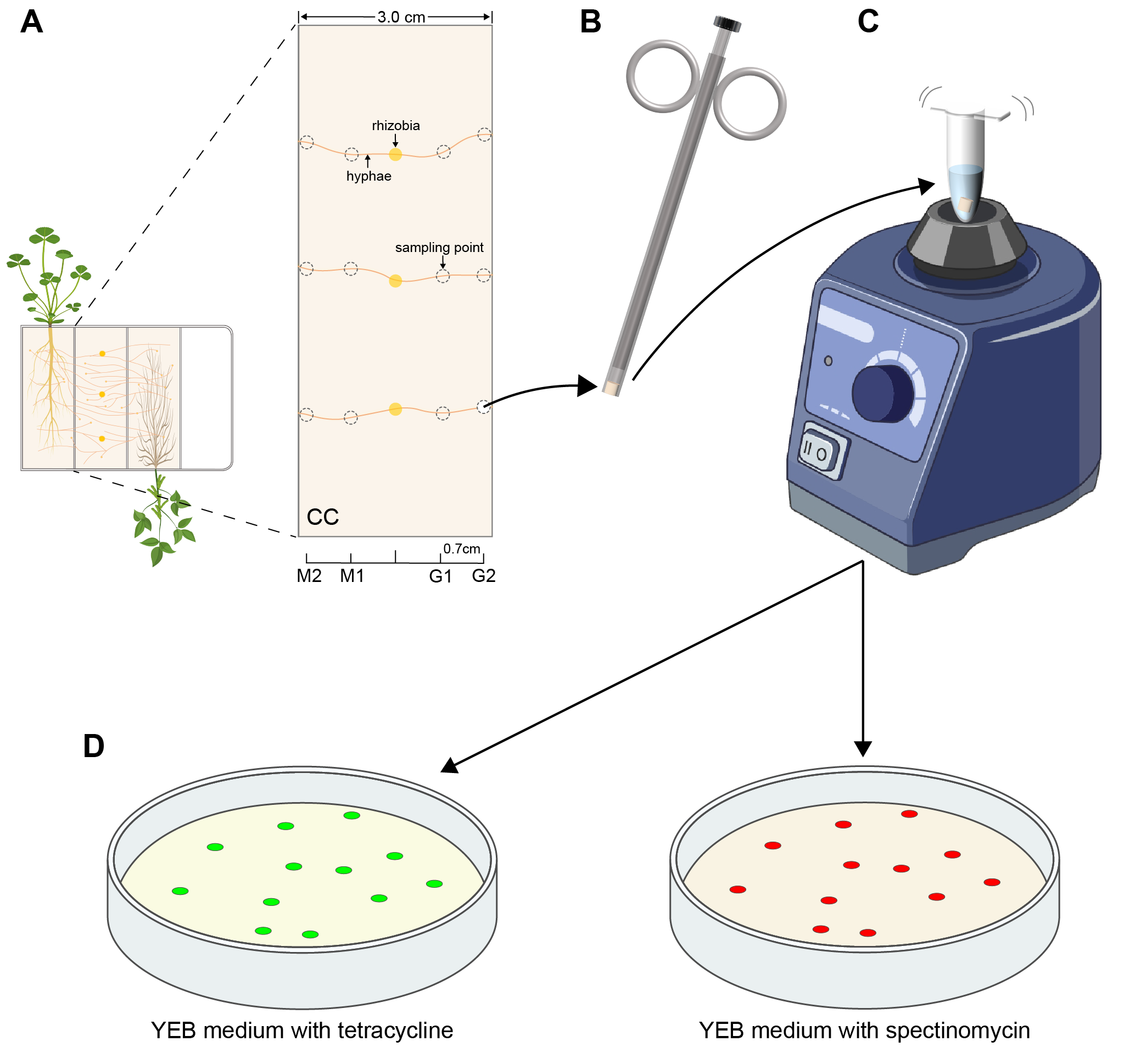


Figure S2. Workflow for sampling and quantifying bacterial migration along hyphae. This figure illustrates the key steps in assessing *S. meliloti* and *B. diazoefficiens* migration along *R. irregularis* hyphae connecting *M. truncatula* and *G. max* within a quadri-compartmented Petri plate system. (A) Diagram of sampling points at 0.7 cm and 1.4 cm from the inoculation site on hyphae in the central compartment. (B) Image of the 2 mm diameter cork borer used to collect medium samples. (C) Schematic of vortexing to homogenize bacterial suspensions from sampled medium. (D) Diagram of selective plating on YEB agar with species-specific antibiotics to enumerate colony-forming units (CFUs).

**Greenhouse experimental design**

**Setup**: A three-compartment pot system (Fig. S3) was designed with a central compartment (CC, 0.3 L) connected to two satellite compartments (0.3 L each) via perforated PVC pipes (1.7 cm diameter × 10 cm length, 6 holes of 6 mm diameter spaced 1 cm apart in the upper portion, Fig. S4). The substrate was a 2:1 (v/v) mixture of sand and vermiculite, autoclaved twice (121°C, 30 min, 0.11 MPa) with a 24 h interval. The pipes were covered with a mesh (5 μm or 41 μm aperture size) at both ends to control hyphal and root passage: the 41 μm mesh allowed only hyphae to proliferate in the pipe, whereas the 5 μm mesh prevented both hyphae and root crossing between compartments. Four treatments were established:

*G.max**≠M.truncatula* treatment: *G. max* and *M. truncatula*, both colonized by *R. irregularis* (5 g inoculum per pot), were grown in the two satellite compartments. Pipes to the CC were covered both side with 5 µm mesh, preventing CMN formation.

*G.max**↔M.truncatula* treatment: *G. max* and *M. truncatula*, both colonized by *R. irregularis* (5 g inoculum per pot), were grown in the satellite compartments. Pipes to the CC were covered both side with 41 µm mesh, enabling a CMN to link the two species in the CC.

*M.truncatula↔M.truncatula* treatment: Two *M. truncatula* plants, both colonized by *R. irregularis* (5 g inoculum per pot), were grown in the satellite compartments. Pipes to the CC were covered both side with 41 µm mesh, enabling a CMN to link the same species.

*G.max↔G.max* treatment: Two *G. max* plants, both colonized by *R. irregularis* (5 g inoculum per pot), were grown in the satellite compartments. Pipes to the CC were covered both side with 41 µm mesh, enabling a CMN to link the same species.


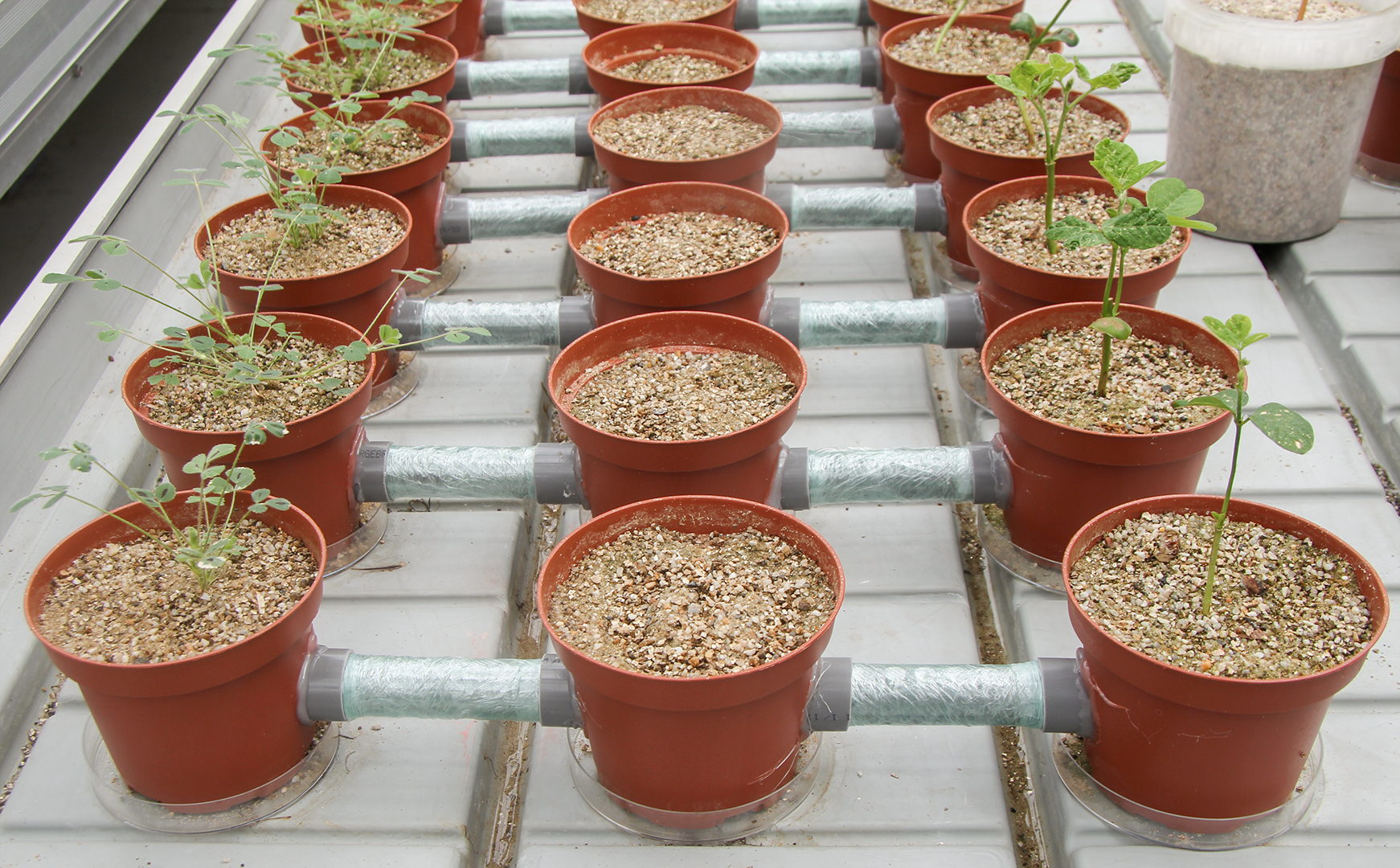


Figure S3. Picture of the three-compartment pot system.


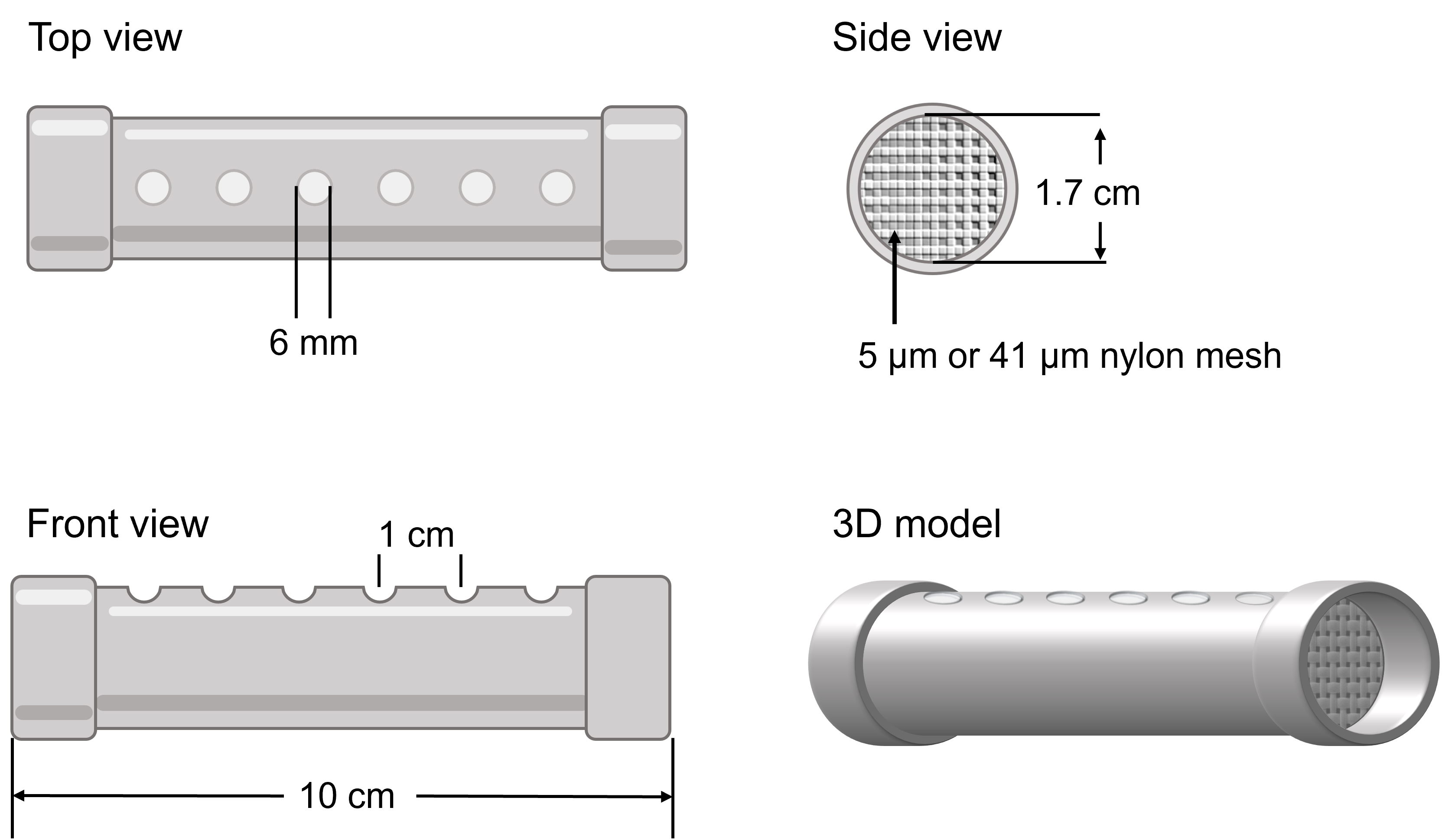


Figure S4. Schematic diagrams of the pipe connected two satellite compartments.

**CMN establishment**: At the start of the experiment, concurrently with the planting and AM fungi inoculation of *M. truncatula* and *G. max* in the satellite compartments, *P. lanceolata* was initially grown in the CC of all pots for four months in the greenhouse to establish the CMN. After this period, the successful establishment of the CMN was confirmed by assessing AM fungal root colonization in roots of *P. lanceolata* from the CC using standard staining [8] and microscopy [9] techniques, and by verifying the presence and measuring the density of fungal hyphae within the connecting pipes using established methods [10]. Following this confirmation, all *P. lanceolata* plants were removed from the CC, and the systems were left undisturbed for an additional month to allow the CMN to stabilize before initiating the experimental treatment phase.

**Inoculation and sampling**: Following the CMN stabilization period, the experimental phase formally began by inoculating the CC with a 5 mL mixture containing *S. meliloti* (2.5 mL, OD_600_ = 1) and *B. diazoefficiens* (2.5 mL, OD_600_ = 1), injected to the center of the substrate surface using a 5 mL NORM-JECT silicone-free Luer Lock Syringe (Henke-Sass Wolf, Germany). To assess rhizobial migration through the CMN, substrate samples were collected from the pipes of three randomly selected systems per treatment at 1, 3, 5, 7, and, 9 days post-inoculation (dpi). Sampling was performed using modified 1 mL pipette tips, cut at 3 cm length from the extremity, inserted into the holes of the pipes; the pipes were then inverted and gently tapped to transfer substrate into 1.5 mL centrifuge tubes (Eppendorf, Germany). For bacterial quantification, samples were mixed with 1 mL PBS, vortexed at 1000 rpm for 20 s, serially diluted 10-fold in PBS, and 20 µL of appropriate dilutions were spread onto selective YEB agar plates (15 g·L^–1^ agar). *S. meliloti* was selected using medium supplemented with 5 µg·mL^–1^ tetracycline, whereas *B. diazoefficiens* was selected with 50 µg·mL^–1^ spectinomycin, following protocols outlined previously [3]. Three biological replicates were considered for each sample. Petri plates were incubated at 28°C for 48 h, and CFUs were counted using an Echo Revolve RVL2-K microscope at 4× magnification, with results converted to CFUs per gram of substrate.

**Harvest and assessment**: At 6 and 8 weeks post-inoculation (wpi), plants were harvested from the satellite compartments of all treatments for various assessments. AM fungal root colonization was evaluated on harvested roots (the same as previously on *P. lanceolata* roots during CMN establishment) under an Olympus BH2-RFCA microscope (Japan) at 10× magnification, scoring 100 intersects per sample.

Soil hyphal density was quantified from fresh substrate samples collected from the pipes. Five grams of fresh substrate was placed in a 50 mL centrifuge tube with 10 mL of 0.1 mol·L^–1^ PBS (pH 7.8). After gentle shaking, 8 mL of the sample solution was transferred to a 10 mL centrifuge tube, followed by the addition of 2 mL of 2% ink (Parker Blue Ink, United States) in 1% HCl. The tube was mixed gently, incubated at 70°C for 20 min, and cooled to room temperature. Then, 1 mL was extracted for microscopic examination (Olympus BH2-RFCA, 10× magnification) to measure hyphal length, following a modified method outlined previously [10]. Three technical replicates per sample and six biological replicates per treatment were used.

Nodule evaluation was also performed, as described above, on harvested roots from satellite compartments.

Rhizobial quantification in the substrate adjacent to the root systems in the satellite compartments was performed at harvest: 5 g substrate samples were collected, and bacterial concentrations were determined using the same extraction, selective plating (YEB with tetracycline for *S. meliloti*, YEB with spectinomycin for *B. diazoefficiens*), incubation, and CFU counting procedures described for the pipe sampling. Three technical replicates and six biological replicates were considered for each sample.

## 1.6. *In silico* binding studies

*In silico* binding studies were performed to evaluate the interaction between selected flavonoids and NodD1 proteins. Eight flavonoids were considered (Table S2), along with NodD1 from *S. meliloti* (UniProtKB-P03031) and *B. diazoefficiens* (UniProtKB-Q45264). Protein sequences were obtained from the UniProt Knowledgebase (<https://www.uniprot.org/help/uniprotkb>). Three-dimensional structures (PDB files) for the NodD1 proteins were predicted using AlphaFold (<https://alphafold.ebi.ac.uk>). The 3D structures of the eight flavonoids were retrieved from PubChem (<https://pubchem.ncbi.nlm.nih.gov>). The NodD1 protein structures and flavonoid ligands were prepared for docking using the AutoDockTools (ADT) package within AutoDock 4.2 [11]. This preparation involved adding polar hydrogen atoms and computing Gasteiger partial charges for both protein and ligand atoms, as well as defining rotatable bonds for the ligands to allow flexibility during docking. Molecular docking was conducted using Autodock Vina (v1.1.2) [12, 13] to form complexes between the NodD1 proteins and flavonoids. The binding affinities of the resulting flavonoid-NodD1 complexes were calculated by Autodock Vina and reported in Kcal·mol^–1^. The docking poses were evaluated based on these predicted affinities, with the most favorable interactions (lowest binding energy) selected for further analysis.

Table S2. The information of eight flavonoids in silico NodD1-ﬂavonoid binding studies

| **No.** | **Flavonoids** | **PubChem CID** | **m/z** |
| --- | --- | --- | --- |
| 1 | DL-Liquiritigenin | 1889 | 255.0656 |
| 2 | Formononetin | 439246 | 273.0756 |
| 3 | Naringenin | 73571 | 287.0910/287.0911 |
| 4 | 3,7-Dimethylquercetin | 5281779 | 329.0660 |
| 5 | Irilone | 5281779 | 299.0548 |
| 6 | Erylatissin A | 11739635 | 353.1382 |
| 7 | Daidzin | 107971 | 417.1180 |
| 8 | 6"-O-Malonyldaidzin | 14500869 | 503.1185 |

# 2. Results


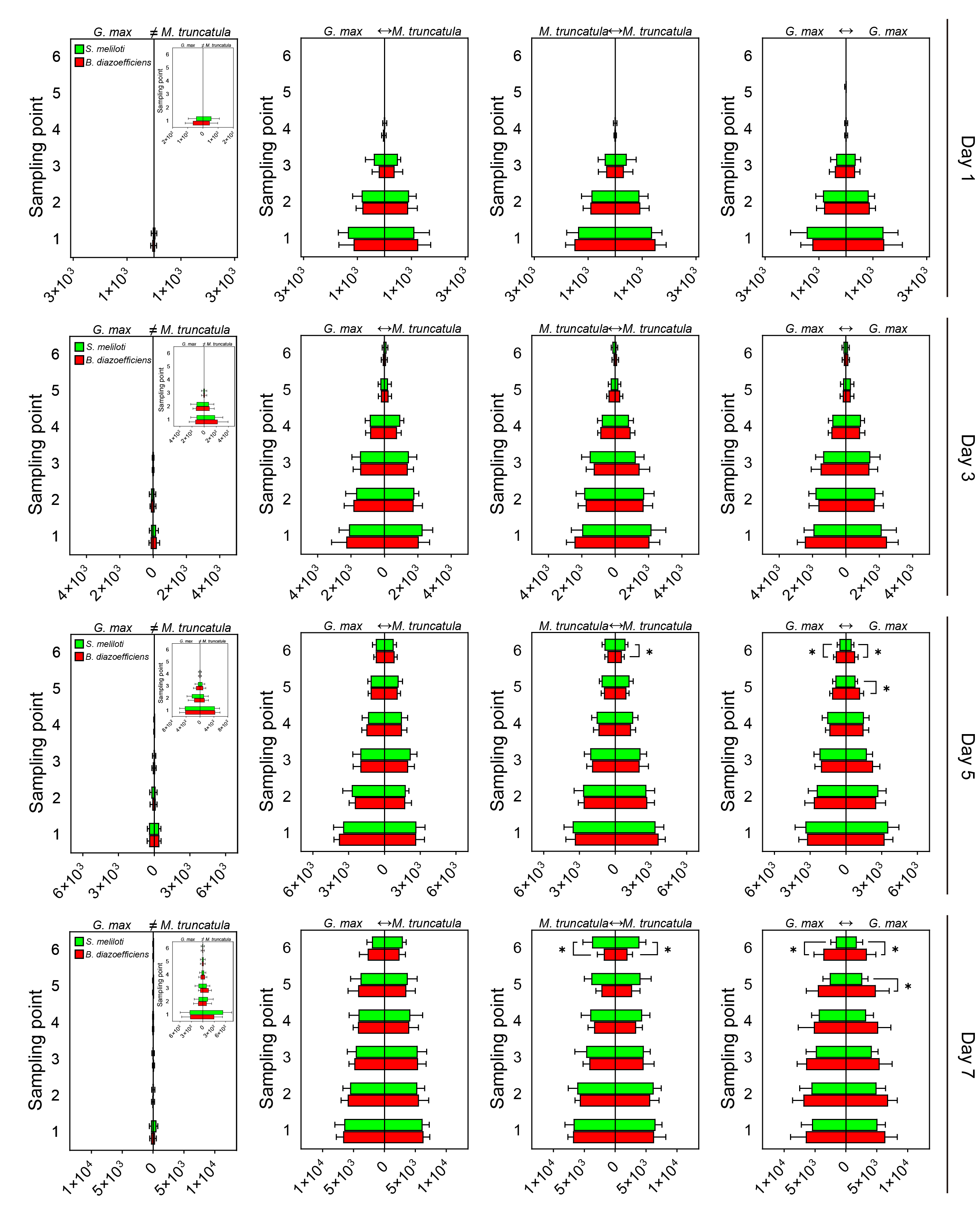


Figure S5. Colony-forming units (CFU·g^–1^ fresh substrate) of *S. meliloti* and *B. diazoefficiens* at different sampling points (holes 1-6, with increasing distance from the CC) in the pipes of *G.max**≠M.truncatula*, *G.max↔M.truncatula*, *M.truncatula↔M.truncatula*, and *G.max↔G.max* treatments at 1, 3, 5, and, 7 days post-inoculation (dpi). Data are means ± SD (*n* = 3 biological replicates, with 3 technical replicates per sample). An asterisk (✱) indicates *P* ≤ 0.05 (Student’s *t*-test for each sampling hole). Where insets are shown for the *G.max≠M.truncatula* treatment (top-right corner of relevant panels), they display the same data as the main panel but with an adjusted x-axis scale to better visualize variations between sampling points for that specific condition.


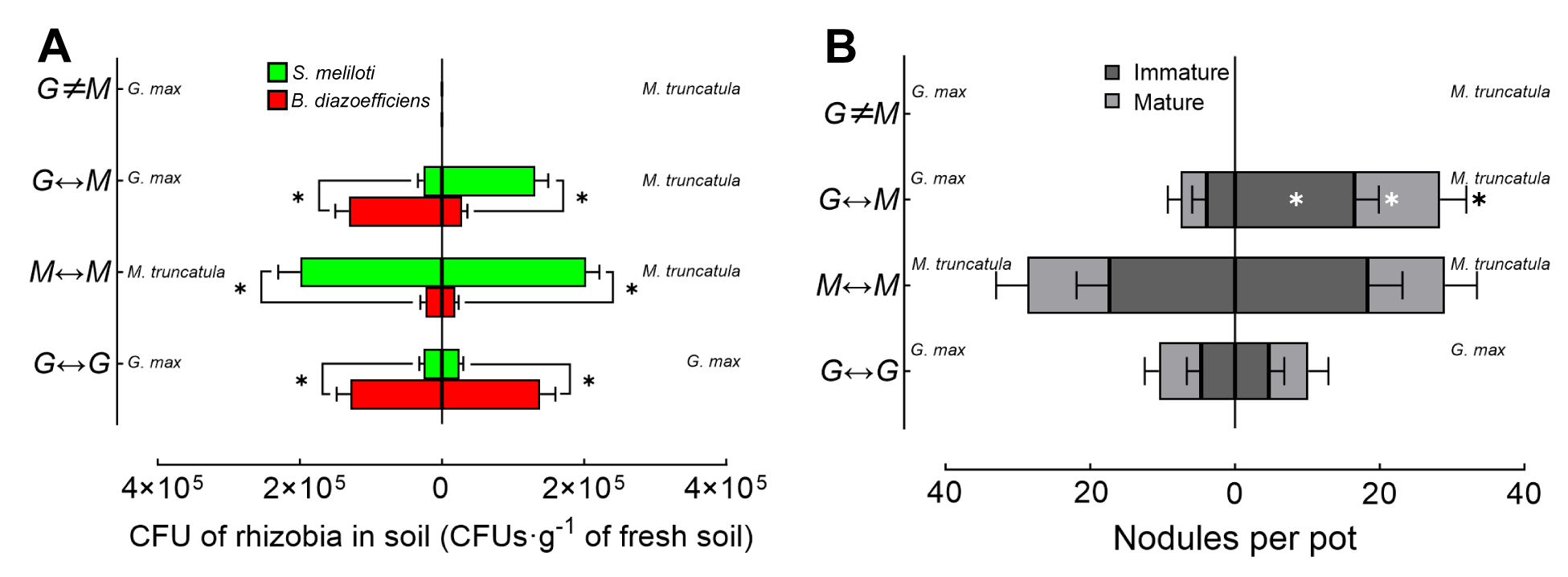


Figure S6. Rhizobial concentrations and nodulation in satellite compartments at 6 weeks post-inoculation (wpi) in greenhouse experiment. (A) Colony-forming units (CFU·g^–1^ fresh substrate) of *S. meliloti* and *B. diazoefficiens* in the satellite compartments in the four treatments at 6 wpi with rhizobia in the CC. Data are means ± SD (*n* = 6 biological replicates, with 3 technical replicates per sample). An asterisk (✱) indicates significant difference (*P* ≤ 0.05, Student’s *t*-test) within each satellite compartment. (B) Numbers of immature and mature nodules on roots of *G. max* and *M. truncatula* in the satellite compartments of the four treatments at 6 wpi with rhizobia in the CC. Data are means ± SD (*n* = 6 biological replicates, with 3 technical replicates per sample). White asterisks (✱) on the bars for immature nodules (dark gray) and mature nodules (light gray) indicate significant differences between the two satellite compartments within each treatment (*P* ≤ 0.05, Student’s *t*-test). Black asterisks (✱) adjacent to the bars denote significant differences in total nodule numbers between the two satellite compartments for each treatment (*P* ≤ 0.05, Student’s *t*-test). G*≠M* (*G.max≠M.truncatula*): *G. max* and *M. truncatula* in satellite compartments connected to the CC via pipes with 5 μm mesh; *G↔M* (*G.max↔M.truncatula*): *G. max* and *M. truncatula* in satellite compartments connected to the CC via pipes with 41 μm mesh; *M↔M* (*M.truncatula↔M.truncatula*): Both satellite compartments with *M. truncatula* and connected to the CC via pipes with 41 μm mesh; *G↔G* (*G.max↔G.max*): Both satellite compartments with *G. max* and connected to the CC via pipes with 41 μm mesh.

# 3. References

1. Declerck S, Strullu DG, Plenchette C. Monoxenic culture of the intraradical forms of *Glomus* sp. isolated from a tropical ecosystem: A proposed methodology for germplasm collection. *Mycologia*. 1998; 90:579–585.

2. Cheng H-P, Walker GC. Succinoglycan is required for initiation and elongation of infection threads during nodulation of alfalfa by *Rhizobium meliloti*. *J Bacteriol*. 1998; 180:5183–5191.

3. He J, Zhang L, Van Dingenen J, Desmet S, Goormachtig S, Calonne-Salmon M, et al. Arbuscular mycorrhizal hyphae facilitate rhizobia dispersal and nodulation in legumes. *ISME J*. 2024; 18:wrae185.

4. Ledermann R, Bartsch I, Remus-Emsermann MN, Vorholt JA, Fischer H-M. Stable fluorescent and enzymatic tagging of *Bradyrhizobium diazoefficiens* to analyze host-plant infection and colonization. *Mol Plant-Microbe Interactions®*. 2015; 28:959–967.

5. Vishwakarma K, Upadhyay N, Kumar N, Verma R, Singh J, Verma P, et al. Microbial interactions in *Litchi* rhizosphere. In: Kumar M, Kumar V, Bhalla-Sarin N, Varma A (eds). *Lychee Disease Management*. 2017. Springer, Singapore, pp 27–44.

6. Saeki K. Rhizobial measures to evade host defense strategies and endogenous threats to persistent symbiotic nitrogen fixation: A focus on two legume-rhizobium model systems. *Cell Mol Life Sci*. 2011; 68:1327–1339.

7. Tsugawa H, Kind T, Nakabayashi R, Yukihira D, Tanaka W, Cajka T, et al. Hydrogen rearrangement rules: Computational MS/MS fragmentation and structure elucidation using MS-FINDER software. *Anal Chem*. 2016; 88:7946–7958.

8. Walker C. A simple blue staining technique for arbuscular mycorrhizal and other root-inhabiting fungi. *Inoculum*. 2005; 56:68–69.

9. McGonigle TP, Miller MH, Evans DG, Fairchild GL, Swan JA. A new method which gives an objective measure of colonization of roots by vesicular-arbuscular mycorrhizal fungi. *New Phytol*. 1990; 115:495–501.

10. Bethlenfalvay GJ, Ames RN. Comparison of two methods for quantifying extraradical mycelium of vesicular-arbuscular mycorrhizal fungi. *Soil Sci Soc Am J*. 1987; 51:834–837.

11. Morris GM, Huey R, Lindstrom W, Sanner MF, Belew RK, Goodsell DS, et al. AutoDock4 and AutoDockTools4: Automated docking with selective receptor flexibility. *J Comput Chem*. 2009; 30:2785–2791.

12. Trott O, Olson AJ. AutoDock Vina: Improving the speed and accuracy of docking with a new scoring function, efficient optimization, and multithreading. *J Comput Chem*. 2010; 31:455–461.

13. Eberhardt J, Santos-Martins D, Tillack AF, Forli S. AutoDock Vina 1.2.0: New Docking Methods, Expanded Force Field, and Python Bindings. *J Chem Inf Model*. 2021; 61:3891–3898.
